# PhWRKY30 activates salicylic acid biosynthesis to positively regulate antiviral defense response in petunia

**DOI:** 10.1093/hr/uhaf013

**Published:** 2025-01-15

**Authors:** Meiling Wang, Yanping Yuan, Yike Zhao, Zhuo Hu, Shasha Zhang, Jianrang Luo, Cai-Zhong Jiang, Yanlong Zhang, Daoyang Sun

**Affiliations:** College of Landscape Architecture and Arts, Northwest A&F University, Yangling, Shaanxi 712100, China; College of Landscape Architecture and Arts, Northwest A&F University, Yangling, Shaanxi 712100, China; College of Landscape Architecture and Arts, Northwest A&F University, Yangling, Shaanxi 712100, China; College of Landscape Architecture and Arts, Northwest A&F University, Yangling, Shaanxi 712100, China; College of Landscape Architecture and Arts, Northwest A&F University, Yangling, Shaanxi 712100, China; College of Landscape Architecture and Arts, Northwest A&F University, Yangling, Shaanxi 712100, China; Department of Plant Sciences, University of California, Davis, Davis, CA 95616, USA; Crops Pathology and Genetics Research Unit, USDA-ARS, Davis, CA 95616, USA; College of Landscape Architecture and Arts, Northwest A&F University, Yangling, Shaanxi 712100, China; College of Landscape Architecture and Arts, Northwest A&F University, Yangling, Shaanxi 712100, China

## Abstract

Petunia (*Petunia hybrida*) plants are highly threatened by a diversity of viruses, causing substantial damage to ornamental quality and seed yield. However, the regulatory mechanism of virus resistance in petunia is largely unknown. Here, we revealed that a member of petunia WRKY transcription factors, *PhWRKY30*, was dramatically up-regulated following Tobacco rattle virus (TRV) infection. Down-regulation of *PhWRKY30* through TRV-based virus-induced gene silencing increased green fluorescent protein (GFP)-marked TRV RNA accumulation and exacerbated the symptomatic severity. In comparison with wild-type (WT) plants, *PhWRKY30*-RNAi transgenic petunia plants exhibited a compromised resistance to TRV infection, whereas an enhanced resistance was observed in *PhWRKY30*-overexpressing (OE) transgenic plants. PhWRKY30 affected salicylic acid (SA) production and expression of *arogenate dehydratase 1* (*PhADT1*), *phenylalanine ammonia-lyase 1* (*PhPAL1*), *PhPAL2b*, *nonexpressor of pathogenesis-related proteins 1* (*PhNPR1*), and *PhPR1* in SA biosynthesis and signaling pathway. SA treatment restored the reduced TRV resistance to WT levels in *PhWRKY30*-RNAi plants, and application of SA biosynthesis inhibitor 2-aminoindan-2-phosphonic acid inhibited promoted resistance in *PhWRKY30*-OE plants. The protein-DNA binding assays showed that PhWRKY30 specifically bound to the promoter of *PhPAL2b*. RNAi silencing and overexpression of *PhPAL2b* led to decreased and increased TRV resistance, respectively. The transcription of a number of reactive oxygen species- and RNA silencing-associated genes was changed in *PhWRKY30* and *PhPAL2b* transgenic lines. PhWRKY30 and PhPAL2b were further characterized to be involved in the resistance to Tobacco mosaic virus (TMV) invasion. Our findings demonstrate that PhWRKY30 positively regulates antiviral defense against TRV and TMV infections by modulating SA content.

## Introduction

To adapt to natural environments with a diverse spectrum of pathogens, plants have evolved two interconnected immune mechanisms: pathogen-triggered immunity (PTI) and effector-triggered immunity (ETI). PTI is initiated by some pattern-recognition receptors localized on cell surface, while ETI is activated by intracellular proteins harboring nucleotide-binding domain leucine-rich repeats [1]. The two mechanisms elicit numerous downstream processes, including the accumulation of reactive oxygen species (ROS), activation of mitogen-activated protein kinases, influx of calcium ions, endogenous hormone synthesis, and transcriptional reprogramming [2, 3]. Plants not only resist pathogen invasion through PTI and ETI at the local site, but also systematically activate and build up broad-spectrum defense against subsequent infection, namely systemic acquired resistance (SAR) [4, 5]. SAR can be initiated by perceiving pathogens via PTI and ETI mechanisms, and this process can be regulated by certain hormones in plants [6, 7].

Salicylic acid (SA) is a key hormone signal to activate PTI, ETI, and SAR for resisting various types of pathogens, including viruses [8]. Many studies have indicated that exogenous SA application enhanced the resistance of plants to some viruses, such as Cucumber mosaic virus (CMV) [9, 10], Cauliflower mosaic virus [11], Potato virus X [12], Tobacco mosaic virus (TMV) [13], Tomato yellow leaf curl virus [14], Mungbean yellow mosaic virus [15], and Sorghum mosaic virus [16]. As the pathogen is recognized by a dominant host resistance gene product, SA production can be promoted in resistant plant hosts [17]. SA was shown to affect antiviral defense through its promoting effect on ROS generation, such as superoxide anion radical (O_2_^−^) and hydrogen peroxide (H_2_O_2_), in mitochondria and inhibiting effect on glyceraldehyde 3-phosphate dehydrogenase [18]. SA-induced increase in ROS levels led to enhanced defense response against viral replication, intercellular dissemination, and systemic movement. This form of signaling was modulated by glutathione and alternative oxidase (AOX) activity [19, 20]. SA can also induce resistance to cell-to-cell movement of viruses through a signaling system that is AOX-independent but less well characterized. Working through nonexpressors of pathogenesis-related proteins 1, 3, and 4 (NPR1, NPR3, and NPR4) and TGA transcription factors (TFs), SA coordinated the activation of a set of *PR* genes and regulated SAR response against nonviral microbial pathogens [21]. SA-elicited increase in the expression of *RNA-dependent RNA polymerase 1* (*RDR1*), a crucial gene of RNA silencing pathway, was also dependent on NPR1. RDR1 was reported to play a crucial role in the maintenance of basal virus resistance [1, 17]. In a different report, SA treatment increased transcript levels of *dicer-like enzyme 1* (*DCL1*), *DCL2*, *RDR1*, and *RDR2* in tomato (*Solanum lycopersicum*) [22]. There seems to be a complex regulatory network involving ROS and RNA silencing that coordinates SA-induced virus resistance. However, the underlying mechanisms of that are not yet fully elucidated.

WRKY proteins comprise a substantial group of zinc finger-type transcriptional regulators, which have been illustrated to play important roles in regulating plant defense response [23]. WRKY proteins are categorized into three classes, mostly based on different primary structures. The members of subgroup I share two C2H2 zinc fingers and two WRKY domains. The members of subgroups II and III possess one WRKY domain, while one C2H2 zinc finger and one C2HC zinc finger are present within the II and III proteins, respectively [24]. The conserved structural domain (WRKYGQK) of WRKY TFs can specifically bind to the W-box (TTGACC/T) *cis*-regulatory element [25]. This W-box motif has been identified in the promoter regions of some genes associated with innate immunity in plants, including PTI, ETI, basal defense, and SAR [26]. The roles of WRKY TFs in defense against virus attack have been partially characterized. For example, GhWRKY15 was shown to positively regulate the resistance of upland cotton (*Gossypium hirsutum*) to TMV and CMV by affecting ROS system [27]. CaWRKYd, a protein from *Capsicum annuum*, was revealed to regulate downstream gene expression, thereby leading to TMV-P0-mediated cell death [23]. AtWRKY8 played an essential role in defense response against crucifer-infecting TMV in Arabidopsis by regulating both abscisic acid (ABA) and ethylene (ET) signaling [28]. NbWRKY40 from *Nicotiana benthamiana* regulated the defense against Tomato mosaic virus (ToMV) by modulating SA signaling [29]. In cassava (*Manihot esculenta*), MeWRKY TFs were involved in the modulation of plant susceptibility to cassava mosaic disease caused by various types of viruses [30].

WRKY30 proteins belong to subgroup III of WRKY family. They have been indicated to have crucial functions in abiotic stress tolerance, including drought, cold, and high salinity [31–33]. Except for abiotic stresses, WRKY30s also play essential roles in modulating various biotic stresses. For example, *Oryza sativa* OsWRKY30 functioned as a transcriptional activator in resistance to the pathogens *Xanthomonas oryzae* pv. *oryzae* and pv. *oryzicola* [34, 35], and the C-terminal region of OsWRKY30 was suggested to be capable of regulating defense signaling [36]. In *Muscadinia rotundifolia*, overexpression of *MrWRKY30* enhanced the resistance to downy mildew pathogen *Peronospora parasitica* [31]. CaWRKY30 might be involved in plant defense mechanisms against diverse pathogen infections in *C. annuum* [37]. Arabidopsis WRKY30 was suggested to function as a positive regulator of plant resistance to CMV infection [38]. There are only a limited number of studies on the roles of WRKY30s in virus resistance. The molecular mechanisms of WRKY30-mediated antiviral defense remain largely unclear.

Petunia (*Petunia hybrida*) is globally recognized as one of the most preferred annual bedding plants due to its abundant, sizable, and vibrant flowers [39]. However, petunia plants are very susceptible to virus infection. A vast quantity of viruses have been reported to infect petunia plants, such as TMV, ToMV, CMV, Tobacco rattle virus (TRV), Impatiens necrotic spot virus, Alfalfa mosaic virus, Potato virus Y, Petunia vein-clearing virus, Tomato ringspot virus, Broad bean wilt I virus, and Petunia chlorotic mottle virus [40, 41]. It has been discovered that these viruses can infect plant cells alone or in combination, resulting in severe symptoms emerged as leaf mosaic, chlorosis, necrosis, deformity, floral discoloration, vein clearing, and plant stunting [42]. These viral pathogens have affected the normal growth and development of petunia, causing considerable economic losses to petunia industry [40]. The current ways preventing petunia virus disease are considered simple and ineffective enough, largely depending on nontoxic seedling propagation, virus vector control, and cultivation management to promote vigorous plant development [43]. Thus, it is necessary to dissect the mechanisms of virus resistance in petunia for antiviral molecular breeding. Among the viruses infecting petunia, TRV, comprising two RNAs (RNA1 and RNA2), is a typical member of the genus *Tobravirus* of family Virgaviridae (Baculovirus). It has a broad range of hosts and high infection efficiency [44]. TRV has been successfully developed as a viral vector in virus-induced gene silencing (VIGS) assay for characterizing gene function [45, 46].

We have employed petunia as a common platform for elucidating the mechanisms of antiviral defense responses. In previous studies, a cluster of differentially expressed genes in petunia plants inoculated with TRV were identified via transcriptome analysis, including many TFs [47]. We have noted that the up-regulated transcripts of WRKY family occupied a relatively high proportion in all TFs with significant differential expression. Of them, a *PhWRKY30* gene was selected for further functional characterization due to its substantial induction in TRV-infected petunia leaves. It has been indicated that several *WRKY30* transcripts from other plant species accumulated rapidly in response to SA treatment [33, 37, 48]. We hypothesize that petunia PhWRKY30 may be implicated in SA-mediated virus resistance. The present work was thus performed to explore the potential role of PhWRKY30 in antiviral defense and SA signaling.

## Results

### PhWRKY30 belongs to subgroup IIIa of WRKY family and possesses transcriptional activity

**Figure 1:**
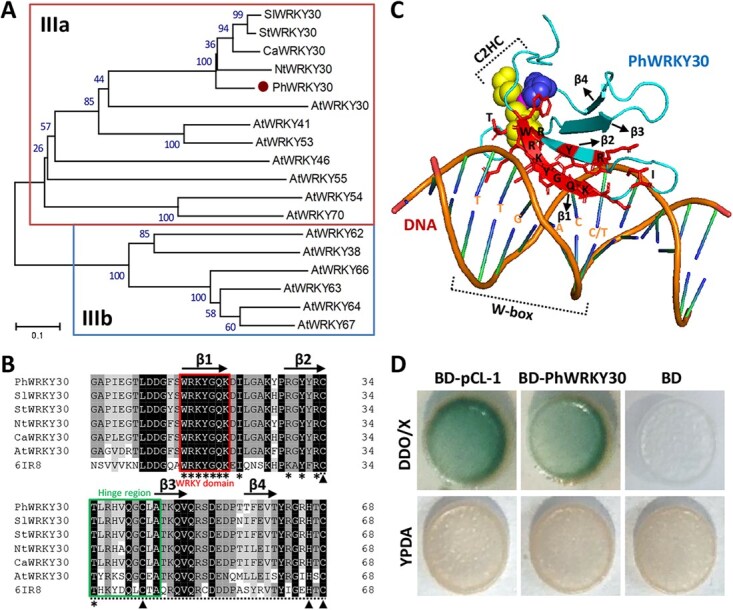
Phylogenetic relationship, sequence structure, and transcriptional activity analysis of PhWRKY30. (**A**) Phylogenetic tree of PhWRKY30 with its similar proteins from *Solanum lycopersicum* (SlWRKY30, XP_004234275), *Solanum tuberosum* (StWRKY30, XP_006350583), *Capsicum annuum* (CaWRKY30, XP_016567798), *Nicotiana tabacum* (NtWRKY30, XP_016512799), and *Arabidopsis thaliana* (AtWRKY30 and other WRKYs). The proteins used for phylogenetic analysis belong to subgroup IIIa and IIIb of WRKY family. Bootstrap values are shown at branch nodes based on the calculation as a percentage of 1000 replicates. PhWRKY30 is denoted by a solid circle. (**B**) Conserved sequence alignment of PhWRKY30 with its homologous proteins from Solanaceae and Arabidopsis plants. The homologous 6IR8 sequence was identified from Protein Data Bank. Four β strands of WRKY30 proteins are shown as arrows. The WRKY domain and hinge region are boxed. Asterisks indicate the important residues for DNA interaction. The zinc finger region is marked by dashed lines, in which three cysteines and one histidine are highlighted by solid triangles. (**C**) Protein modeling of PhWRKY30 for the potential DNA-binding interaction. The residues binding to W-box motif (TTGACC/T) are shown as sticks. The spheres represent the C2HC-type zinc finger region. (**D**) Transcriptional activation of PhWRKY30 in yeast cells. The pCL-1 was used as a positive control, while the BD EV was used as a negative control. The blue cells represent positive transcriptional activation.

To investigate the function of WRKY TFs in response to viruses, one up-regulated transcript, *PhWRKY30* (SGN accession no. Peaxi162Scf00904g00212), was identified from TRV-infected leaf transcriptome data in petunia (Supplementary Fig. S1) [47]. The full-length *PhWRKY30* cDNA contains a 1056-bp coding region, encoding a polypeptide of 352 amino acids (Supplementary Fig. S2). Phylogenetic analysis showed that PhWRKY30 is highly similar to other WRKY30s from *S. lycopersicum*, *S. tuberosum*, *C. annuum*, *Nicotiana tabacum*, and *Arabidopsis thaliana* (Fig. 1A), suggesting that PhWRKY30 is a putative member of subgroup IIIa of WRKY family. Conserved sequence alignment of these WRKY30s with a homologous protein structure (Protein Data Bank: 6IR8) was performed. As shown in Fig. 1B, PhWRKY30 harbors a conserved WRKY domain, a hinge region, and a CX7CX23HXC (C2HC)-type zinc finger motif. Four typical β strands are present within this sequence of PhWRKY30. Protein structural modeling of PhWRKY30 revealed that 11 residues (WRKYGQK, R, Y, R, and T) in close proximity to the groove of DNA molecule have the binding potential to W-box motif (TTGACC/T) (Fig. 1C). To test the transcriptional activity of PhWRKY30, a yeast-based transactivation assay was carried out. The yeast cells transformed with BD-PhWRKY30 were shown in blue on the SD/*-His-Trp* medium containing X-α-gal, similar to that observed for the positive control BD-pCL-1 (Fig. 1D). This indicates that PhWRKY30 may serve as a transcriptional activator.

### *PhWRKY30* is induced by TRV infection and hormone treatments

**Figure 2:**
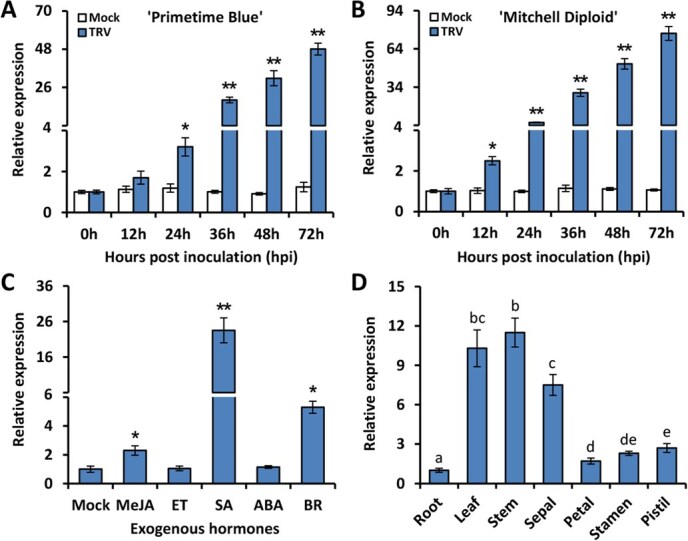
Expression of *PhWRKY30* in petunia leaves under virus infection and hormone treatments and in different tissues. Reverse transcription-quantitative PCR (RT-qPCR) analysis of expression levels of *PhWRKY30* in the leaves from petunia cultivar ‘Primetime Blue’ (**A**) and ‘Mitchell Diploid’ (**B**) plants at different hours postinoculation (hpi) with TRV (PPK20). The inoculation with deionized water was used as mock control. (**C**) RT-qPCR analysis of expression levels of *PhWRKY30* in ‘Mitchell Diploid’ leaves treated with 100 μM methyl jasmonate (MeJA), 10 μl L^−1^ ethylene (ET), 100 μM SA, 50 μM abscisic acid (ABA), and 50 μM BR. The treatment with deionized water was used as mock control. (**D**) Expression of *PhWRKY30* in various tissues of ‘Mitchell Diploid’ plants through RT-qPCR analysis. Four-leaf-stage petunia seedlings were used for inoculation or treatment. Different samples used for tissue-specific expression analysis were harvested from 8-week-old petunia plants in the flowering period. *PhEF1α* was used to normalize expression data. Error bars represent standard error of the mean from three biological replicates. Asterisks or different letters suggest statistical significance based on the calculation by Student’s *t*-test (^*^P < 0.05, ^**^P < 0.01) or one-way ANOVA test (*P* < 0.05), respectively.

To verify the up-regulation of *PhWRKY30* by TRV infection, reverse transcription-quantitative PCR (RT-qPCR) analysis was carried out to examine transcript profiles of *PhWRKY30* in two petunia cultivars ‘Primetime Blue’ and ‘Mitchell Diploid’. Compared to mock control, a continuous increase in expression levels of *PhWRKY30* was found in the leaves of both cultivars from 0 to 72 h postinoculation (hpi) with TRV. In contrast, the cultivar ‘Mitchell Diploid’ displayed a higher induction of *PhWRKY30* than ‘Primetime Blue’ at each time point (Fig. 2A and B). Given the interplays between plant hormones and virus invasion [49], we examined the impacts of several antivirus-related hormones on the transcription of *PhWRKY30*. The results showed that *PhWRKY30* was remarkably up-regulated following treatments with methyl jasmonate (MeJA), SA, and brassinosteroid (BR), of which SA application led to the highest induction. No significant alteration in transcript levels of *PhWRKY30* was observed after ET and ABA treatments (Fig. 2C). *PhWRKY30* was expressed in leaves, stems, and sepals at relatively higher levels than in roots and floral tissues including petals, stamens, and pistils (Fig. 2D). These results indicate that PhWRKY30 may be implicated in hormone-associated anti-TRV defense response.

### Virus-induced *PhWRKY30* silencing promotes TRV accumulation

**Figure 3:**
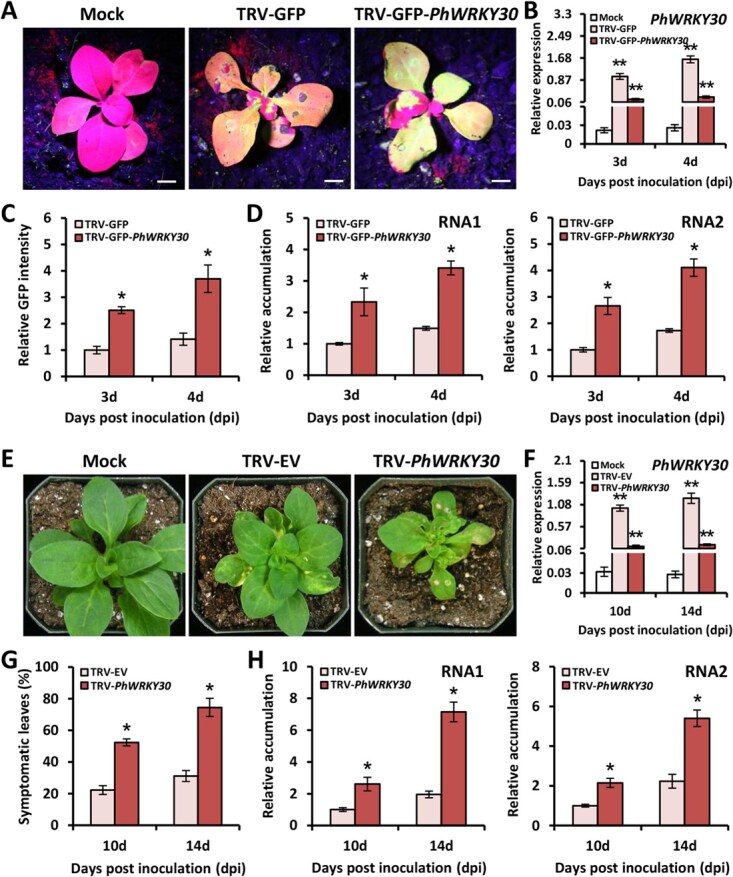
Silencing of *PhWRKY30* through virus-induced gene silencing system reduces resistance to TRV infection. (**A**) Green fluorescent protein (GFP) signals in the leaves from petunia cultivar ‘Primetime Blue’ plants inoculated with *Agrobacterium* bearing TRV-GFP and TRV-GFP-*PhWRKY30*. The inoculation with deionized water was used as mock control. The plants at 4 dpi were photographed under UV light. Scale bars = 5 mm. (**B**) Reverse transcription-quantitative PCR (RT-qPCR) analysis of expression levels of *PhWRKY30* in mock-, TRV-GFP-, and TRV-GFP-*PhWRKY30*-infected leaves at 3 and 4 dpi. Relative GFP intensities (**C**) and accumulation levels of TRV RNAs (RNA1 and RNA2) (**D**) in the inoculated leaves at different time points. (**E**) Symptoms of petunia cultivar ‘Primetime Blue’ plants inoculated with *Agrobacterium* bearing TRV EV and TRV-*PhWRKY30*. The inoculation with deionized water was used as mock control. Photographs were taken using the plants at 14 dpi. (**F**) RT-qPCR analysis of expression levels of *PhWRKY30* in mock-, TRV-EV-, and TRV-*PhWRKY30*-infected leaves at 10 and 14 dpi. (**G**) Percentage of symptomatic leaves in the plants infected with TRV constructs. (**H**) Relative accumulation levels of TRV RNAs in systemically infected leaves. *PhEF1*α was used as the reference gene for expression analysis. Error bars indicate standard error of the mean from three biological replicates. Asterisks suggest significance of difference as determined by Student’s *t*-test (^*^P < 0.05, ^**^P < 0.01).

To determine the role of PhWRKY30 in defense against TRV infection, a TRV-based VIGS system was employed to knockdown its transcripts. The fragment of *PhWRKY30* was firstly cloned into a TRV vector expressing green fluorescent protein (GFP), which served as a marker for TRV propagation. At 4 days postinoculation (dpi), petunia leaves inoculated with TRV-GFP-*PhWRKY30* exhibited more fluorescent foci than TRV-GFP-infected ones. No fluorescent signal was detected in mock control leaves (Fig. 3A). RT-qPCR analysis showed that expression levels of *PhWRKY30* in TRV-GFP-*PhWRKY30*-infected leaves were markedly lower than those in TRV-GFP-infected ones at 3 and 4 dpi. Both TRV-GFP and TRV-GFP-*PhWRKY30* infections led to higher transcript levels of *PhWRKY30* than mock control (Fig. 3B). Correspondingly, silencing of *PhWRKY30* resulted in increased GFP intensities and accumulation levels of TRV RNAs (RNA1 and RNA2) in the leaves compared to TRV-GFP (Fig. 3C and D). To examine the difference in viral symptoms, we used a TRV empty vector (EV) bearing the *PhWRKY30* fragment for inoculation. TRV-*PhWRKY30*-infected petunia plants showed enhanced leaf curling and necrosis and inhibited growth at 14 dpi in comparison with TRV-EV and mock control (Fig. 3E). The leaves infected with TRV-EV and TRV-*PhWRKY30* showed increased expression of *PhWRKY30* than mock control at 10 and 14 dpi. A substantial down-regulation of *PhWRKY30* in TRV-*PhWRKY30*-infected leaves was confirmed by RT-qPCR in comparison to TRV-EV (Fig. 3F). Similarly, *PhWRKY30*-silenced plants displayed higher percentage of symptomatic leaves and TRV accumulation levels than nonsilenced control (Fig. 3G and H), suggesting that silencing of *PhWRKY30* reduces petunia resistance to TRV attack.

### RNAi silencing and overexpression of *PhWRKY30* affect susceptibility to TRV infection

**Figure 4:**
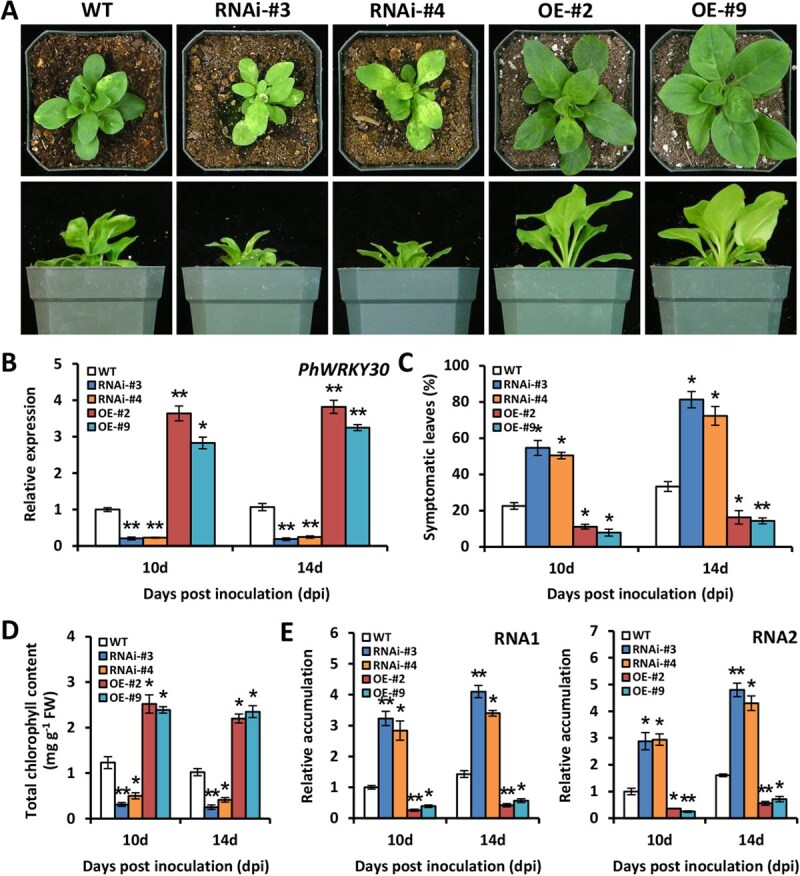
RNAi silencing and overexpression of *PhWRKY30* affect resistance to TRV infection. (**A**) Symptoms of WT, *PhWRKY30*-RNAi (#3 and #4), and *PhWRKY30*-overexpressing (OE) (#2 and #9) transgenic lines of petunia cultivar ‘Mitchell Diploid’ inoculated with TRV (PPK20). The plants at 14 dpi were photographed at top view and side view. (**B**) Reverse transcription-quantitative PCR (RT-qPCR) analysis of expression levels of *PhWRKY30* in uppermost systemically infected leaves from WT, *PhWRKY30*-RNAi, and *PhWRKY30*-OE transgenic petunia lines at 10 and 14 dpi. (**C**) Percentage of symptomatic leaves in WT and transgenic petunia plants infected with TRV. (**D**) Total chlorophyll content and (**E**) relative accumulation levels of TRV RNAs (RNA1 and RNA2) in systemically infected leaves from WT and transgenic petunia lines. Expression or accumulation levels were standardized to *PhEF1*α. Error bars indicate standard error of the mean from three biological replicates. Statistical significance was evaluated according to Student’s *t*-test (^*^P < 0.05, ^**^P < 0.01) and denoted by asterisks.

To corroborate the findings in VIGS assay, we generated *PhWRKY30*-RNAi and *PhWRKY30*-overexpressing (OE) transgenic petunia plants through stable genetic transformation. PCR analysis validated the integration of *PhWRKY30* gene into the genome of a number of positive transgenic lines (Supplementary Fig. S3). Two RNAi lines (#3 and #4) and OE lines (#2 and #9) were selected for functional characterization. In comparison with wild-type (WT) plants, *PhWRKY30*-RNAi transgenic petunia lines exhibited more severe leaf mottling and chlorosis and plant stunting at 14 dpi with TRV, whereas *PhWRKY30*-OE lines had much milder viral symptoms with nonmosaic leaves and normal growth (Fig. 4A). RT-qPCR analysis showed that expression levels of *PhWRKY30* were reduced by 77%–88% in two RNAi lines and increased by 1.8–2.8-folds in two OE lines at 10 and 14 dpi (Fig. 4B), validating the silencing and overexpression of *PhWRKY30* in transgenic plants. Consistent with symptom appearance, RNAi silencing of *PhWRKY30* caused increased symptomatic leaf ratio in petunia plants infected with TRV, while decreased ratio was found in the plants overexpressing *PhWRKY30* (Fig. 4C). *PhWRKY30*-RNAi lines showed lower total chlorophyll content and higher accumulation levels of TRV RNA1 and RNA2 than WT line, but its overexpression caused an opposite influence on the accumulation of total chlorophyll and TRV RNAs (Fig. 4D and E). These data demonstrate that PhWRKY30 functions as a positive regulator of defense against TRV invasion.

### PhWRKY30 participates in SA-mediated antiviral defense response

**Figure 5:**
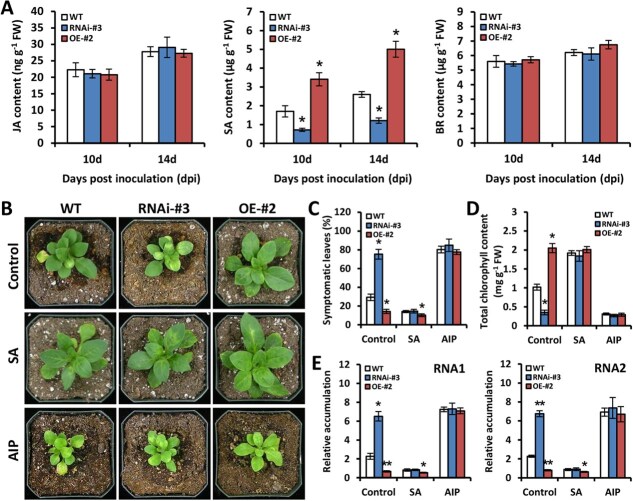
PhWRKY30 is involved in SA-mediated defense response against virus infection. (**A**) Contents of JA, SA, and BR in the leaves from WT, *PhWRKY30*-RNAi (#3), and *PhWRKY30*-overexpressing (OE) (#2) transgenic lines of petunia cultivar ‘Mitchell Diploid’ inoculated with TRV (PPK20). The leaf samples at 10 and 14 dpi were harvested for measurement of endogenous hormone levels. (**B**) Symptoms of WT and transgenic petunia lines treated with 100 μM SA and 100 μM 2-aminoindan-2-phosphonic acid (AIP), an inhibitor of SA biosynthesis. The water treatment was used as the control. Photographs were taken using the plants at 14 dpi. (**C**) Percentage of symptomatic leaves in TRV-infected WT and transgenic petunia plants treated with SA and AIP at 14 dpi. (**D**) Total chlorophyll content and (**E**) relative accumulation levels of TRV RNAs (RNA1 and RNA2) in systemically infected leaves from WT and transgenic petunia lines under SA and AIP treatments at 14 dpi. Accumulation levels were normalized to the reference gene *PhEF1*α. Error bars represent standard error of the mean from three biological replicates. Significance of difference was calculated according to Student’s *t*-test (*P < 0.05, **P < 0.01) and denoted by asterisks.

Considering the up-regulation of *PhWRKY30* by exogenous MeJA, SA, and BR treatments, we measured the production of these hormones in TRV-infected WT and *PhWRKY30* transgenic petunia plants. As shown in Fig. 5A, SA levels were significantly lower in *PhWRKY30*-RNAi line (#3) and higher in *PhWRKY30*-OE line (#2) than those in WT line at 10 and 14 dpi. However, silencing and overexpression of *PhWRKY30* did not affect the contents of JA and BR. Thus, we speculate that PhWRKY30 probably regulates defense response against TRV infection through the SA pathway.

To test this hypothesis, exogenous SA was used to treat WT and *PhWRKY30* transgenic petunia plants infected with TRV. Compared with nontreated control, SA treatment enhanced the resistance of WT, *PhWRKY30*-RNAi, and *PhWRKY30*-OE transgenic lines to TRV at 14 dpi. Especially, SA-treated WT and *PhWRK30*-RNAi lines showed similar viral symptoms. By comparison, application of 2-aminoindan-2-phosphonic acid (AIP), an SA biosynthesis inhibitor [50], decreased virus resistance of all tested lines, which displayed almost identical symptoms (Fig. 5B). WT and *PhWRKY30*-silenced lines after SA application exhibited insignificant change in symptomatic leaf ratio, total chlorophyll content, and TRV accumulation levels. These values were much similar in WT and transgenic lines following AIP treatment (Fig. 5C-E). Moreover, we reduced the SA production in *PhWRKY30*-OE plants by silencing *phenylalanine ammonia-lyase 1* (*PhPAL1*), a key SA biosynthetic gene [51], using VIGS system. It was found that TRV-*PhPAL1*-infected *PhWRKY30*-OE plants had similar viral symptom and TRV accumulation to TRV-EV-infected WT plants (Supplementary Fig. S4). These results indicate that SA treatment restored the compromised TRV resistance of *PhWRKY30*-silenced plants to WT levels, and SA deficiency repressed the enhanced TRV resistance of *PhWRKY30*-OE plants. We therefore conclude that PhWRKY30 regulates resistance to TRV by modulating SA content.

### PhWRKY30 binds to the promoter of SA biosynthesis-related *PhPAL2b*

**Figure 6:**
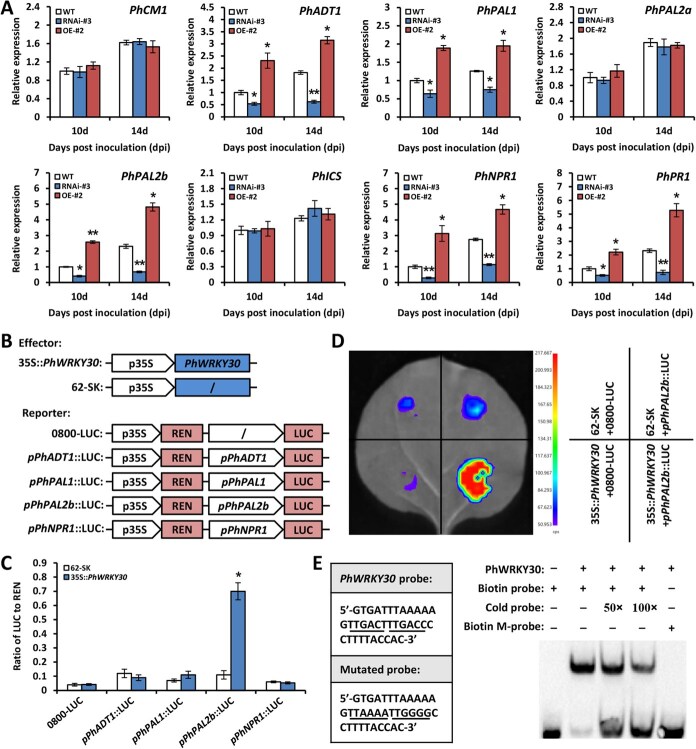
PhWRKY30 specifically transactivates the promoter of *PhPAL2b*. (**A**) Reverse transcription-quantitative PCR (RT-qPCR) analysis of expression levels of SA biosynthesis-associated genes, including *PhCM1*, *PhADT1*, *PhPAL1*, *PhPAL2a*, *PhPAL2b*, *PhICS*, *PhNPR1*, and *PhPR1*, in the leaves from WT, *PhWRKY30*-RNAi (#3), and *PhWRKY30*-overexpressing (OE) (#2) transgenic lines of petunia cultivar ‘Mitchell Diploid’ inoculated with TRV (PPK20). The leaf samples at 10 and 14 dpi were harvested for expression analysis. (**B**) Schematic diagrams of the effector and reporter constructs for dual luciferase (LUC) assay. p35S, 35S promoter; *pPhADT1*, *pPhPAL1*, *pPhPAL2b*, and *pPhNPR1*, promoters of *PhADT1*, *PhPAL1*, *PhPAL2b*, and *PhNPR1*; LUC, firefly luciferase; REN, *Renilla* luciferase. (**C**) Dual LUC assay of *PhADT1*, *PhPAL1*, *PhPAL2b*, and *PhNPR1* promoters with PhWRKY30. Transactivation activities are expressed as LUC/REN. (**D**) Transactivation of *PhPAL2b* promoter by PhWRKY30. The *Agrobacteria* bearing different constructs were co-infiltrated into *Nicotiana benthamiana* leaves. Co-infiltrations of 62-SK with 0800-LUC, 62-SK with *pPhPAL2b*::LUC, and 35S::*PhWRKY30* with 0800-LUC served as negative controls. The LUC images were taken at 3 days postinfiltration. (**E**) The binding of PhWRKY30 to a biotin-labeled probe of *PhPAL2b* promoter through electrophoretic mobility shift assay (EMSA). Two W-box *cis*-elements and their mutated nucleotides are underlined. 50- and 100-fold concentrations of nonlabeled probes (cold) were regarded as competitors. *PhEF1*α was used as the reference gene for expression analysis. Error bars indicate standard error of the mean from three biological replicates. Asterisks denote significance of difference as evaluated by Student’s *t*-test (^*^P < 0.05, ^**^P < 0.01).

To better elucidate the role of PhWRKY30 in the regulation of SA pathway, the transcription of a couple of genes related with SA biosynthesis and signaling was examined by RT-qPCR. Expression levels of SA biosynthetic genes *arogenate dehydratase 1* (*PhADT1*), *PhPAL1*, and *PhPAL2b* as well as SA signaling genes *PhNPR1* and *PhPR1* were lower in *PhWRKY30*-RNAi line (#3) than those in WT control, but higher in transgenic line (#2) overexpressing *PhWRKY30*. Transcript abundances of other biosynthesis-related genes *chorismate mutase 1* (*PhCM1*), *PhPAL2a*, and *isochorismate synthase* (*PhICS*) were not changed by *PhWRKY30* silencing and overexpression (Fig. 6A).

To identify the candidate target genes of PhWRKY30, we analyzed the *cis*-elements in the promoters of above differentially expressed genes. It has been revealed that Arabidopsis AtWRKY30 binds to consensus W-box motif (TTGACC/T) with high specificity and affinity [32]. Various numbers of W-box motifs were found in the promoters of *PhADT1*, *PhPAL1*, *PhPAL2b*, and *PhNPR1* in sense or antisense orientation (Supplementary Fig. S5). Coding region of *PhWRKY30* and these promoters were used to generate the effector and reporter constructs (Fig. 6B). According to that, a dual luciferase assay was then conducted. The activity of firefly luciferase (LUC) was increased by 6.4 times when co-expressing 35S::*PhWRKY30* and *pPhPAL2b*::LUC compared to the control (62-SK) (Fig. 6C). Enhanced LUC fluorescent signals were visualized for the PhWRKY30-*pPhPAL2b* interaction (Fig. 6D), suggesting a transactivation of *PhPAL2b* promoter by PhWRKY30. To determine whether PhWRKY30 could bind to W-box motif, a 36-bp fragment harboring both W-box *cis*-elements (TTGACT and TTGACC) in the *PhPAL2b* promoter was amplified as the probe. Electrophoretic mobility shift assay (EMSA) showed that PhWRKY30 clearly bound to the biotin-labeled probe rather than the nucleotide substituted mutant. This binding was inhibited when adding 50- and 100-fold nonlabeled probes (Fig. 6E). These findings suggest that PhWRKY30 directly binds to the promoter of *PhPAL2b*.

### RNAi silencing and overexpression of *PhPAL2b* affect susceptibility to TRV infection

**Figure 7:**
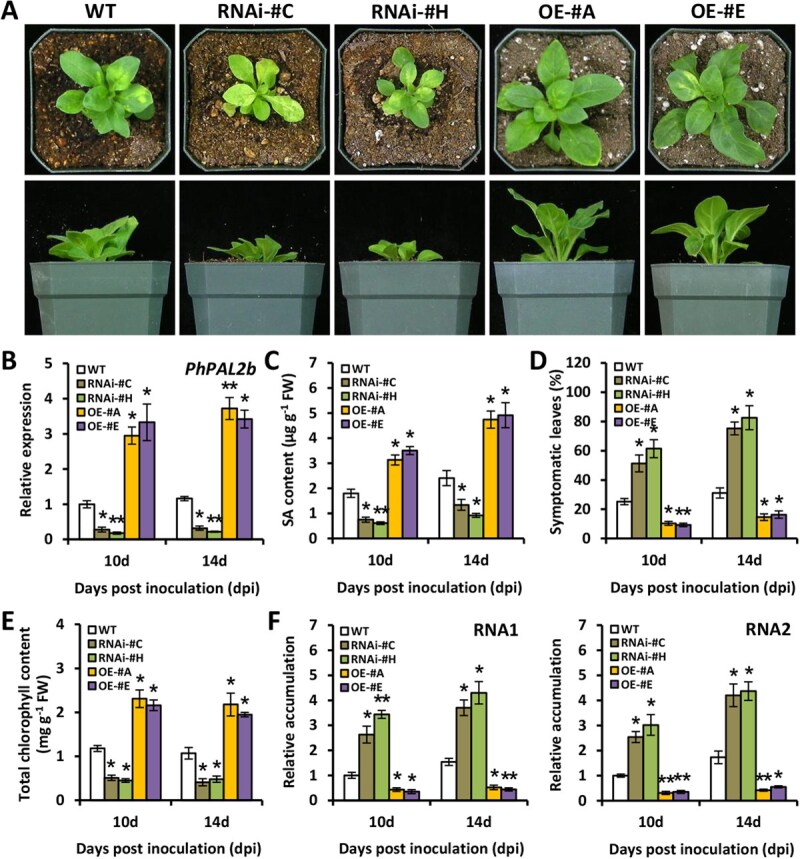
RNAi silencing and overexpression of *PhPAL2b* affect resistance to TRV infection. (**A**) Symptoms of WT, *PhPAL2b*-RNAi (#C and #H), and *PhPAL2b*-overexpressing (OE) (#A and #E) transgenic lines of petunia cultivar ‘Mitchell Diploid’ inoculated with TRV (PPK20). The plants at 14 dpi were photographed at top view and side view. (**B**) Reverse transcription-quantitative PCR (RT-qPCR) analysis of expression levels of *PhPAL2b* in uppermost systemically infected leaves from WT, *PhPAL2b*-RNAi, and *PhPAL2b*-OE transgenic petunia lines at 10 and 14 dpi. (**C**) Content of SA in TRV-infected leaves from WT and transgenic petunia lines. (**D**) Percentage of symptomatic leaves in WT and transgenic petunia plants infected with TRV. (**E**) Total chlorophyll content and (**F**) relative accumulation levels of TRV RNAs (RNA1 and RNA2) in systemically infected leaves from WT and transgenic petunia lines. Expression or accumulation levels were normalized to the reference gene *PhEF1*α. Error bars suggest standard error of the mean from three biological replicates. Statistical significance was evaluated by Student’s *t*-test (^*^P < 0.05, ^**^P < 0.01) and indicated by asterisks.

To further study the function of PhPAL2b in antiviral defense, *PhPAL2b* was down-regulated and up-regulated by RNAi and overexpression assays, respectively. The introduction of transgene into petunia genome was determined through PCR amplification (Supplementary Fig. S6), and we chose several RNAi and OE lines for functional analysis. At 14 dpi with TRV, two *PhPAL2b*-RNAi lines (#C and #H) showed more severe viral symptoms, and relatively milder symptoms were observed in two *PhPAL2b*-OE lines (#A and #E) in comparison with WT line (Fig. 7A). The reduced and increased transcription of *PhPAL2b* in RNAi and OE lines were validated by RT-qPCR (Fig. 7B). SA content was lower in the plants with *PhPAL2b* silencing and higher in the ones with *PhPAL2b* overexpression (Fig. 7C). When checking the ratio of symptomatic leaves, we found that the RNAi and OE lines displayed higher and lower ratios than WT control, respectively (Fig. 7D). A decline in total chlorophyll content and a rise in accumulation levels of TRV RNAs were detected in *PhPAL2b*-RNAi plants, while overexpression of *PhPAL2b* led to elevated chlorophyll content and decreased TRV RNA levels (Fig. 7E and F). These observations indicate that PhPAL2b plays an essential role in regulating resistance to TRV infection.

**Figure 8:**
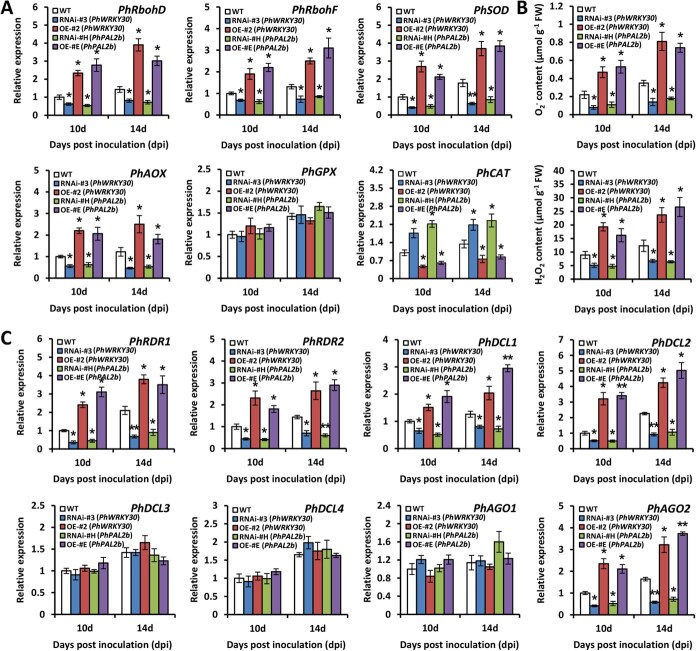
PhWRKY30 and PhPAL2b regulate the expression of ROS- and RNA silencing-related genes. (**A**) Reverse transcription-quantitative PCR (RT-qPCR) analysis of expression levels of genes associated with ROS generation (*PhRbohD*, *PhRbohF*, and *PhSOD*) and ROS scavenging (*PhAOX*, *PhGPX*, and *PhCAT*) in uppermost systemically infected leaves from WT, *PhWRKY30-*RNAi (#3), *PhWRKY30-*overexpressing (OE) (#2), *PhPAL2b-*RNAi (#H), and *PhPAL2b-*OE (#E) transgenic petunia lines inoculated with TRV (PPK20). The leaf samples at 10 and 14 dpi were harvested for expression assessment. (**B**) Levels of O_2_^−^ and H_2_O_2_ in TRV-infected leaves from WT and *PhWRKY30* and *PhPAL2b* transgenic petunia lines at 10 and 14 dpi. (**C**) RT-qPCR analysis of expression levels of RNA silencing pathway genes, including *PhRDR1*, *PhRDR2*, *PhDCL1*, *PhDCL2*, *PhDCL3*, *PhDCL4*, *PhAGO1*, and *PhAGO2*, in TRV-infected leaves from WT and *PhWRKY30* and *PhPAL2b* transgenic petunia lines. *PhEF1α* served as an internal control. Error bars represent standard error of the mean from three biological replicates. Asterisks suggest significance of difference, which was analyzed by Student’s *t*-test (^*^P < 0.05, ^**^P < 0.01).

### PhWRKY30 and PhPAL2b modulate the expression of genes associated with ROS and RNA silencing

It has been reported that SA-induced virus resistance is associated with ROS and RNA silencing pathways [17, 52]. To explore whether PhWRKY30 and its downstream PhPAL2b regulate the two pathways, we tested the transcription of some genes involved in ROS generation and scavenging and RNA silencing at 10 and 14 dpi with TRV. Based on RT-qPCR analysis, we found that expression levels of *respiratory burst oxidase homolog D* (*PhRbohD*), *PhRbohF*, and *superoxide dismutase* (*PhSOD*) related with ROS production and *PhAOX*, a negative regulator of ROS formation, decreased in *PhWRKY30*- and *PhPAL2b*-RNAi transgenic petunia lines compared to WT line, and their expression levels increased in *PhWRKY30*- and *PhPAL2b*-OE lines. On the contrary, the transcription of ROS-scavenging gene *catalase* (*PhCAT*) increased in *PhWRKY30*- and *PhPAL2b*-RNAi lines and decreased in their OE lines (Fig. 8A). The RNAi lines of *PhWRKY30* and *PhPAL2b* exhibited lower levels of O_2_^−^ and H_2_O_2_, two main types of ROS, than WT plants, whereas the OE lines of *PhWRKY30* and *PhPAL2b* showed higher O_2_^−^ and H_2_O_2_ levels (Fig. 8B). Besides, a few RNA silencing-related genes, including *PhRDR1*, *PhRDR2*, *PhDCL1*, *PhDCL2*, and *argonaute 2* (*PhAGO2*), were down-regulated and up-regulated in RNAi and OE lines of *PhWRKY30* and *PhPAL2b*, respectively (Fig. 8C). The results suggest that PhWRKY30 probably regulates SA-induced virus resistance relying on ROS generation and RNA silencing.

### PhWRKY30 and PhPAL2b are involved in defense response against TMV infection

**Figure 9:**
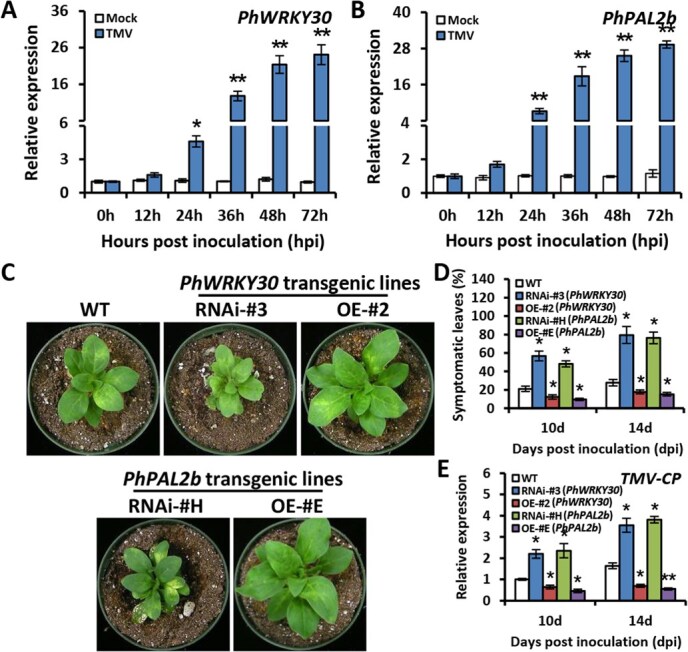
PhWRKY30 and PhPAL2b are involved in resistance to TMV infection. Reverse transcription-quantitative PCR (RT-qPCR) analysis of expression levels of *PhWRKY30* (**A**) and *PhPAL2b* (**B**) in the leaves from petunia cultivar ‘Mitchell Diploid’ plants at different hpi with TMV (U1). The inoculation with deionized water was used as mock control. (**C**) Symptoms of WT, *PhWRKY30-*RNAi (#3), *PhWRKY30-*overexpressing (OE) (#2), *PhPAL2b-*RNAi (#H), and *PhPAL2b-*OE (#E) transgenic petunia lines inoculated with TMV. The plants at 14 dpi were photographed. (**D**) Percentage of symptomatic leaves in WT and *PhWRKY30* and *PhPAL2b* transgenic petunia plants infected with TMV. (**E**) RT-qPCR analysis of expression levels of *TMV coat protein* (*TMV-CP*) in TMV-infected leaves from WT and *PhWRKY30* and *PhPAL2b* transgenic petunia lines. Transcript levels were standardized to the reference gene *PhEF1α*. Error bars indicate standard error of the mean from three biological replicates. Asterisks suggest statistical significance based on the calculation by Student’s *t*-test (^*^P < 0.05, ^**^P < 0.01).

Given the crucial role of SA in plant resistance to multiple viruses, the function of PhWRKY30 and PhPAL2b in response to TMV infection was then analyzed. In comparison with mock control, TMV infection caused a dramatic and continuous increase in transcript abundances of *PhWRKY30* and *PhPAL2b* (Fig. 9A and B). At 14 dpi with TMV, *PhWRKY30*- and *PhPAL2b*-RNAi lines showed aggravated symptoms of leaf mottling and deformation compared to WT control, but the alleviated symptom development was observed in *PhWRKY30*- and *PhPAL2b*-OE lines. RNAi silencing and overexpression of *PhWRKY30* and *PhPAL2b* inhibited and promoted the growth of plants upon TMV infection, respectively (Fig. 9C). In line with that, the RNAi lines of *PhWRKY30* and *PhPAL2b* had higher symptomatic leaf ratios than WT line, while their OE lines had lower ratios (Fig. 9D). *PhWRKY30*- and *PhPAL2b*-silenced lines had more transcripts of *TMV-CP*, encoding TMV coat protein, but fewer *TMV-CP* transcripts were accumulated in their OE lines (Fig. 9E). These data demonstrate that PhWRKY30 may confer a broad-spectrum virus resistance by modulating SA production.

## Discussion

**Figure 10:**
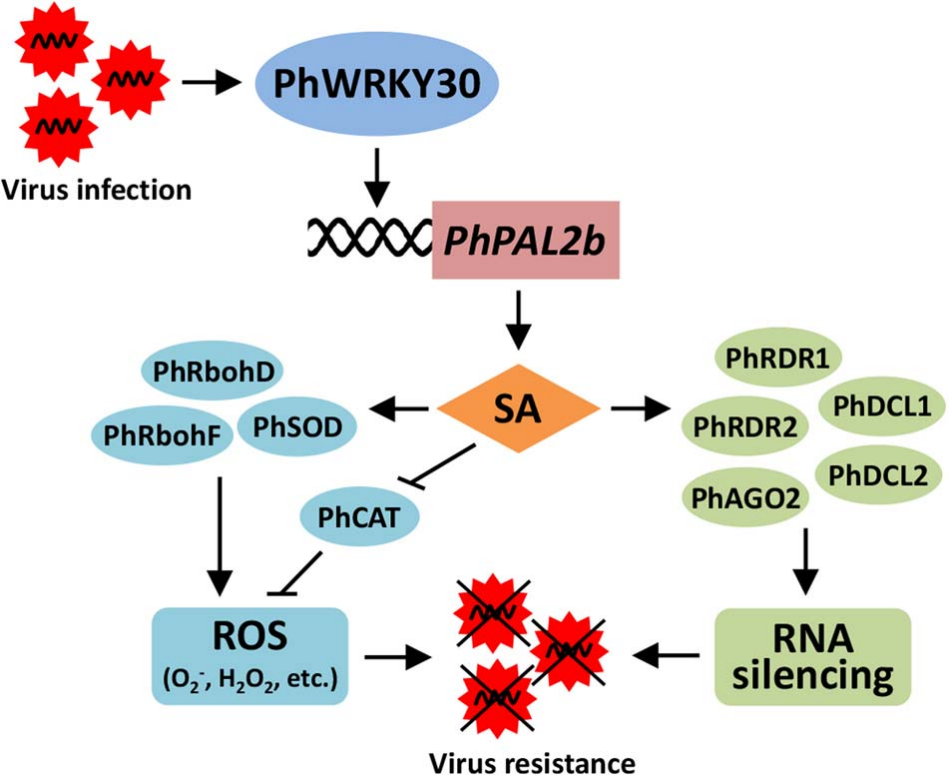
A proposed model for the role of PhWRKY30 in antiviral defense response. The infection with TRV or TMV induces the transcription of *PhWRKY30*. PhWRKY30 specifically regulates the downstream gene *PhPAL2b* by binding to its promoter, resulting in promoted SA biosynthesis. SA contributes to virus resistance by modulating ROS production and RNA silencing pathways. Solid line ending with an arrow or a short perpendicular line indicates a positive or a negative regulation, respectively.

Viruses account for almost 47% of the pathogens that trigger emerging and re-emerging plant diseases worldwide, posing a substantial threat to crop growth and production [53]. It is widely recognized that the use of molecular breeding to obtain virus-resistant varieties is the most economic and effective method to mitigate virus-triggered losses [54]. Therefore, the elucidation of molecular regulatory mechanisms of antiviral defense in plants is required. SA is an important hormone signal in plant defense against virus attack. However, how SA-induced virus resistance is regulated by the TFs is not yet fully understood. In this study, we uncovered the pivotal role of a petunia WRKY TF, PhWRKY30, in the regulation of antiviral response. PhWRKY30 contributed to plant resistance to TRV and TMV infections by directly activating a SA biosynthetic gene *PhPAL2b*, which was revealed to play a similar antiviral role as PhWRKY30 (Figs 4-7, and 9). The transcription of some genes associated with ROS accumulation and RNA silencing was affected by both PhWRKY30 and PhPAL2b (Fig. 8). Altogether, we propose a model for elucidating the involvement of PhWRKY30-PhPAL2b module in antiviral defense by targeting the SA pathway (Fig. 10).

### PhWRKY30 functions as a positive regulator of antiviral response in petunia

Based on phylogenetic analysis, PhWRKY30 falls into subgroup IIIa of WRKY family (Fig. 1A). Many members of this subgroup have been demonstrated to be associated with plant responses to a variety of pathogens. For example, SA-induced Arabidopsis WRKY46 was identified to modulate basal resistance to *Pseudomonas syringae* in collaboration with WRKY70 and WRKY53 [55]. Ectopic overexpression of *VqWRKY52* from *Vitis quinquangularis* enhanced resistance to powdery mildew and *P. syringae* rather than *Botrytis cinerea* in Arabidopsis [56]. ShWRKY41 was shown to confer resistance to *Oidium neolycopersici* by reducing the production of H_2_O_2_ and hypersensitive response in wild tomato (*Solanum habrochaites*) [57]. GhWRKY53 affected the tolerance of upland cotton (*G. hirsutum*) to *Verticillium dahliae* by mediating SA and JA signaling [58]. However, Arabidopsis WRKY70 and its similar protein WRKY54 played negative roles in defense responses to *Pectobacterium carotovorum* and *B. cinerea* by regulating cell wall fortification [59]. It appears that most of previous studies have focused on the roles of subgroup IIIa TFs in responses to fungal pathogens. However, there are few studies on the regulation of viral pathogens by the members of subgroup IIIa in plants. Although WRKY30 has been suggested to confer resistance to CMV in Arabidopsis, the detailed regulatory mechanism underlying WRKY30-mediated antiviral defense remains still elusive. Our data here suggest a novel role of subgroup IIIa TFs of WRKY family in defense against TRV and TMV infections.

Numerous differentially expressed transcripts have been identified in plants inoculated with viruses through RNA sequencing (RNA-Seq) method [60–63]. In a previous study, we have performed a high-throughput RNA-seq analysis on TRV-infected petunia leaves [47], and the transcript *PhWRKY30* was thus screened out due to its dramatic up-regulation. This up-regulation was further confirmed here by RT-qPCR analysis in two petunia cultivars ‘Primetime Blue’ and ‘Mitchell Diploid’, implying the essential role of PhWRKY30 in regulating TRV resistance. Our present results validated the involvement of PhWRKY30 in anti-TRV defense response (Figs 3 and 4). Apart from *PhWRKY30*, dozens of WRKY TFs also showed variable expression in TRV-infected leaf transcriptome data, with up-regulated TFs accounting for the majority [47]. It appears likely that multiple WRKY TFs individually or cooperatively contribute to defense response against TRV invasion in petunia. Further research should be performed to identify the important antiviral genes from those up-regulated WRKY TFs using a rapid screening tool, such as VIGS.

By comparison, the cultivar ‘Mitchell Diploid’ showed higher expression levels of *PhWRKY30* than the cultivar ‘Primetime Blue’ at each time point of TRV infection (Fig. 2A and B). It may partially explain the discrepancy in virus resistance among petunia cultivars. It has been indicated that gene expression variation can distinguish the resistant and susceptible cultivars in response to viral pathogens [64, 65]. According to our observations, the ‘Mitchell Diploid’ plants are more resistant to virus attack than the ‘Primetime Blue’ plants, which is in accordance with relatively higher transcription in the former. That is why we commonly used ‘Primetime Blue’ but not ‘Mitchell Diploid’ for TRV-based VIGS assays [45, 66, 67], to ensure adequate virus accumulation and high gene silencing efficiency in petunia. As a homozygous inbred cultivar, ‘Mitchell Diploid’ seems to be more suitable as one of the parents for antiviral crossbreeding. It also can serve as a good model for identifying the key virus-resistant genes and further dissecting the molecular genetic basis of antiviral immunity in petunia.

### PhWRKY30 participates in antiviral defense by modulating SA-mediated ROS generation and RNA silencing

SA can repress viral propagation at different phases of its infection, including viral replication, intercellular spread, and long-distance movement [18]. Endogenous SA content has been suggested to be closely correlated with antiviral capacity of various plant species, such as tobacco (*N. tabacum*) [68], Arabidopsis [69], potato (*S. tuberosum*) [70], sour organge (*Citrus tristeza*) [71], maize (*Zea mays*) [72], and cassava (*M. esculenta*) [73]. In line with this notion, we previously found that SA production and expression of a set of SA biosynthetic genes increased in petunia leaves following TRV inoculation [47]. Here, application of SA resulted in a higher induction of *PhWRKY30* expression compared to other hormones (Fig. 2C), suggesting a potential regulation of SA by PhWRKY30 during TRV infection. This is supported by altered transcription of *PhPAL2b* in *PhWRKY30* transgenic lines and specific interaction between PhWRKY30 and *PhPAL2b* promoter (Fig. 6). It was shown that transcript levels of *PhPAL2b* decreased in *PhWRKY30*-RNAi line (#3) and increased in *PhWRKY30*-OE line (#2) compared to WT plants at 10 and 14 dpi with TRV (Fig. 6A). Our further expression analysis determined that *PhPAL2b* was also down-regulated and up-regulated in the other RNAi line (#4) and OE line (#9) of *PhWRKY30*, respectively (Supplementary Fig. S7). It is worth mentioning that PhWRKY30 bound to the promoter fragment of *PhPAL2b* bearing two types of W-box motifs (TTGACC and TTGACT) (Fig. 6C-E). It remains unsolved which motif possesses higher binding activity for this interaction. It has been suggested that the sequence variance of WRKY domain, zinc finger region, and adjacent zone of core motifs could affect WRKY/W-box recognition specificity [74]. A further promoter binding activity analysis should be performed to determine the binding preference of PhWRKY30 to certain W-box motifs.

Although the promoters of *PhADT1*, *PhPAL1*, and *PhNPR1* contain consensus W-box motifs, PhWRKY30 had no binding activity to these promoters through dual luciferase assay (Fig. 6C). This implies that PhWRKY30 may have variable preferences for transcriptional activation of different promoters bearing W-box motifs. One of the possible explanations is that the nucleotide sequence of the core motif is often not sufficient to ensure a successful TF-DNA binding, which is normally dependent on additional factors beyond the binding sites [75–77]. Thus, some crucial elements flanking the core W-box motif probably exist to be required for the direct binding of PhWRKY30 to *PhPAL2b* promoter in vivo. A further site analysis may be essential for fully understanding PhWRKY30-bound DNA shape features. In addition, the interaction of PhWRKY30 with unknown proteins may also lead to the differences of binding preferences. This is supported by some previous studies in which certain protein–protein interactions weakened or impaired sequence-dependent DNA binding specificities of TFs [78, 79]. The impact of PhWRKY30-interacting protein on its DNA binding activities should be examined in future work.

Some lines of evidence have revealed the connection of SA-induced virus resistance with ROS signals. SA can interfere with the respiratory electron transport chain, resulting in increased production of ROS in mitochondria [80]. Moderate levels of ROS have proven to function as secondary messengers of signal transduction pathways and play protective roles in response to viral attack [81]. A virus-derived small interference RNA, vsiRNA1, was reported to positively regulate ROS production, and its expression in *Triticum aestivum* conferred broad-spectrum resistance to Wheat yellow mosaic virus, Barley stripe mosaic virus, and Chinese wheat mosaic virus [82]. Silencing of *NbLHCB3*, encoding light-harvesting chlorophyll a/b complex protein 3, caused over-accumulation of ROS and reduced susceptibility to Turnip mosaic virus infection in *N. benthamiana* [83]. In this study, we found that ROS-producing genes *PhRbohD*, *PhRbohF*, and *PhSOD* were down-regulated and up-regulated in *PhWRKY30*- and *PhPAL2b*-RNAi and their OE lines, respectively. An opposite expression trend of RNA-scavenging gene *PhCAT* in transgenic lines was observed (Fig. 8A). This suggests that SA promotes ROS accumulation and inhibits ROS elimination, thereby eliciting the accumulation of O_2_^−^ and H_2_O_2_ (Fig. 8B). It is an undeniable fact that excess ROS production can bring about oxidative damage to lipids, DNAs, and proteins, finally resulting in cell death and necrosis symptom appearance [84]. It has been reported that Alpha-momorcharin (α-MMC), a member of the plant ribosomal inactivating protein family, was implicated in defense against TMV infection by up-regulating ROS-scavenging transcripts and reducing ROS accumulation [85]. The contradictory findings demonstrate that ROS probably plays a complex role in plant–virus interaction, most likely depending on the concentrations of ROS. We conclude that it is quite essential to keep a dynamic balance in ROS-mediated immune response. Our results showed that expression levels of *PhAOX*, a negative modulator of ROS generation, decreased in RNAi lines of *PhWRKY30* and *PhPAL2b* and increased in the OE lines (Fig. 8A). This possibly acts as a negative feedback loop in SA-induced ROS accumulation, facilitating the detoxification of ROS to maintain redox homeostasis.

Another important mechanism governing plant resistance to viruses is RNA silencing, which is a sequence-specific RNA degradation process [86]. Foreign viral RNA molecules can be targeted for destruction by RNA silencing machinery, which requires the components RDRs, DCLs, and AGOs [87]. In the present study, expression analysis showed that several components, including *PhRDR1*, *PhRDR2*, *PhDCL1*, *PhDCL2*, and *PhAGO2*, were positively regulated by PhWRKY30 and PhPAL2b (Fig. 8C). These are almost in agreement with previous reports on the induction of *RDR*, *DCL*, or *AGO* genes in tomato (*S. lycopersicum*) [22] and petunia [46] following SA treatment. These results indicate a link between RNA silencing and SA-mediated virus resistance. However, an earlier study showed that a few RNA silencing-related proteins, such as *DCL2*, *DCL3*, and *DCL4*, were not crucial for SA-induced CMV and TMV resistance [9]. In contrast, some SA-related TFs, belonging to MYB, bZIP, and WRKY families, were reported to co-express with a few *RDR*, *DCL*, and *AGO* genes, whose promoters contain multiple binding sites of these identified TFs [88]. The defense response of sour orange to Citrus tristeza virus (CTV) was modulated by both SA and RNA silencing pathways, and CTV p20 and p23, two RNA silencing suppressors, impaired SA signaling defense [71]. A recent report revealed that a SA-activated RDR prevented virus proliferation in plant stem cells [89]. Accordingly, it is highly likely that SA-induced antiviral response is partially dependent on RNA silencing, which in turn reinforce SA-mediated virus resistance. A complete regulatory network connecting SA and RNA silencing in defense against viruses should be dissected in future work.

### PhWRKY30 probably plays multiple roles in plant immunity and physiology by modulating SA production

It is well known that SA is regarded as an inducer of SAR in plants, contributing to the activation of defense responses against various biotic stresses. In addition to viruses, SA was shown to be closely associated with plant resistance to distinct bacteria [90, 91], fungi [92, 93], oomycetes [94, 95], and nematodes [96]. Differing from SA-mediated antiviral mechanism, it has been demonstrated that a couple of PR proteins, such as PR1, PR2, and PR5, in SA signaling pathway are responsible for the resistance to nonviral pathogens [97, 98]. Our data showed that transcript levels of *PhPR1* were reduced in transgenic petunia plants with *PhWRKY30* silencing, and increased in *PhWRKY30*-OE plants (Fig. 6A), suggesting a possible contribution of PhWRKY30 to the resistance to other microbial pathogens. In support of this notion, both inoculations with *P. syringae* and *B. cinerea* up-regulated the transcription of *PhWRKY30* in petunia leaves (Supplementary Fig. S8). Some homologs of PhWRKY30, including *C. annuum* CaWRKY30 [37], *O. sativa* OsWRKY30 [36], and *M. rotundifolia* MrWRKY30 [31], have been identified as important regulators of anti-bacterial or anti-fungal responses. Whether PhWRKY30 participates in petunia resistance to diverse pathogens requires further examination in subsequent studies.

Increasing evidence has verified the roles of SA in plant tolerance to abiotic stresses, like drought, high salinity, heat, cold, and heavy metals [99]. The mechanisms underlying these SA-mediated tolerances vary in responses to different abiotic stresses. For instance, SA was found to increase the tolerance of safflower (*Carthamus tinctorius*) to salt by promoting the accumulation of carbohydrates, glycine betaines, chlorophylls, pigments, and total soluble proteins [100]. SA improved wheat yield and yield-related traits under drought condition by enhancing chlorophyll content and membrane stability index [101]. Despite the revealed function of WRKY30s in tolerance to salt, drought, and heat in some plant species [31–33], how SA mediates these tolerance responses is still unclear. It is plausible that PhWRKY30 may play a broad role in the regulation of abiotic factors relying on the SA pathway. Future studies should include a thorough investigation of PhWRKY30’s roles in responses to other environmental stimuli.

In addition, the importance of SA signals in the modulation of plant growth and development cannot be ignored. Many Arabidopsis mutants with overproduction of SA presented dwarf phenotypes with smaller leaves or flowers and shorter stems [102, 103]. By comparison, SA-deficient Arabidopsis mutant showed increased leaf biomass and seed yield [104]. In agreement with that, we found that both *PhWRKY30*- and *PhPAL2b*-RNAi transgenic lines displayed increased plant height compared to WT line, whereas their OE lines exhibited inhibited growth (Supplementary Fig. S9). However, a controversial finding revealed that the rice *abnormal inflorescence meristem1* (*aim1*) mutant with decreased SA content had shorter seedling and adventitious roots [105]. It has been reported that a 3-mM concentration of SA was most effective in improving postharvest fruit quality of *Prunus persica* [106]. SA at lower dozes resulted in the optimal seed germination performance and shoot elongation of teosinte (*Z. mays* ssp. *mexicana*), but these effects were reduced at higher dozes [107]. We thus hypothesize that SA plays multifaceted roles in plant growth and development depending on its concentration and plant species. Furthermore, it is noteworthy that expression levels of *PhWRKY30* increased by MeJA and BR (Fig. 2C), which also function as regulators of plant growth and defense against external unfavorable factors [108, 109]. Whether a crosstalk between SA and these two hormones exists in PhWRKY30-regulated petunia growth or stress responses requires further investigation.

In conclusion, our data suggest that PhWRKY30 functions as a positive regulator of resistance to TRV and TMV infections by modulating SA content. The theoretical foundation of molecular regulatory mechanism underlying virus resistance is thus enriched. The findings presented here will be helpful to control virus disease through molecular genetic engineering in petunia. Due to the targeting of SA pathway, PhWRKY30 was presumed to have a wide range of roles in plant growth and development and responses to various environmental factors. In line with this presumption, the rice OsWKRY45, similar to PhWRKY30, showed a zinc-mediated dimerization at the hinge region [110], and this structural feature was considered to widen the spectrum of protein function [111]. An extensive dissection on the regulatory roles of PhWRKY30 would be made in future work.

## Materials and methods

### Plant materials and growth conditions

In this study, we used two petunia (*P. hybrida*) cultivars, obtained from Goldsmith Seed Company (Gilroy, CA, USA), as experimental materials. The cultivar ‘Primetime Blue’ was used for transiently down-regulating gene expression through TRV-based VIGS assay, while the cultivar ‘Mitchell Diploid’ was employed to generate stable RNAi and OE transgenic petunia plants. These petunia seeds were germinated in a 96-well plastic tray with a transparent cover containing the soil mixture composed of peat moss, vermiculite, and perlite in a volumetric ratio of 1:1:1, and then transferred to square pots for the follow-up cultivation. They were maintained in an environment-controlled chamber with a temperature of 26/22°C (day/night), relative humidity of 75%, and a cycle of 16/8 hours (light/dark). Four-leaf-stage petunia plants were used for *Agrobacterium* infiltration, virus inoculation, or exogenous hormone treatments. Upper healthy leaves were harvested as explants for stable genetic transformation. Young roots, leaves, stems, and floral tissues (sepals, petals, stamens, and pistils) on the first day of flowering of 8-week-old ‘Mitchell Diploid’ plants were harvested as samples for gene expression analysis in various tissues.

### Identification of PhWRKY30

The cDNA sequence of *PhWRKY30*, which contains a complete open reading frame (ORF) with 1056 bp in length, was isolated using PCR method. Its nucleotides were translated into amino acids using ExPASy tool (http://web.expasy.org/translate/). Homologous sequence comparison of PhWRKY30 with the other proteins was carried out via DNAMAN software (version 8.0). Phylogenetic tree was established using MEGA4 program (version 4.0.2). The C2HC zinc finger and WRKY domain were identified according to a previous study [24]. A homologous structure (6IR8) of PhWRKY30 was obtained from the RCSB Protein Data Bank (https://www.rcsb.org/). The protein modeling analysis of PhWRKY30’s conserved region was conducted using Modeller tool (version 9.20). The model was ultimately visualized and analyzed using PyMOL software (version 2.5.4). The important residues, which are responsible for DNA interaction or dimerization are denoted by sticks or spheres.

### Transactivation activity analysis

The transactivation activity of PhWRKY30 was assessed based on a yeast system. The coding region of *PhWRKY30*, excluding the stop codon, was amplified using the forward primer bearing *Nde*I site and reverse primer bearing *Sal*I site (Supplementary Table S1). The amplified sequence was then introduced into the pGBKT7 vector to create the fusion construct pGBKT7-*PhWRKY30*. The resulting construct was introduced into the yeast (*Saccharomyces cerevisiae*) strain Y2HGold according to a protocol of user manual (Takara, Otsu, Shiga, Japan). The pCL1 was used as a positive control for activity analysis, whereas the pGBKT7 EV served as a negative control. The transformants were cultured on the medium SD/*−Trp* and SD/*-His-Trp* at 30°C for a period of 3 to 5 days to compare the cell proliferation and blue color appearance. Three independent experiments were performed for this analysis.

### Virus inoculation assay

The wild TRV strain PPK20 and TMV strain U1 preserved in *N. tabacum* cultivar ‘NC82’ plantlets were used in this study. The preparation of infectious agent and inoculation procedure were performed following a previous description [47] with slight modifications. Infectious sap was produced by grinding infected young leaves in a solution of 100 mM phosphate buffer with a small amount of kieselguhr at pH 7.0 (1:6, w/v). Petunia seedlings at four-leaf stage with uniform growth were used for inoculation. The viral solution mixed with carborundum powder (600 mesh) was rubbed onto the surface of fully expanded leaves using a sterile glass spreader. A gentle rubbing with at least three times was carried out to guarantee a sufficient virus entry into leaf cells. After 20 min, the inoculated leaves were flushed using sterile deionized water to eliminate extra inoculum and further dried with absorbent paper. The symptoms caused by TRV and TMV inoculation were observed, and relative accumulation levels of viruses were examined during the postinoculation periods. Three biological replicates were used with three seedlings in each replicate.

### Exogenous hormone treatments

To study the effects of antivirus-associated hormones on the transcription of *PhWRKY30*, the plantlets of petunia cultivar ‘Mitchell Diploid’ were treated by MeJA, ET, SA, ABA, and BR at different concentrations. Application of ET was conducted through a continuous exposure of petunia seedlings to 10 μl L^−1^ gaseous ET in a sealed chamber. The treatments with remaining hormones were carried out via foliar spraying with the solutions of 100 μM MeJA, 100 μM SA, 50 μM ABA, and 50 μM BR, respectively. The spraying was applied until both sides of the leaves were fully wetted. To examine the impact of SA treatment on PhWRKY30-mediated virus resistance, WT, *PhWRKY30*-RNAi, and *PhWRKY30*-OE transgenic lines at 2 dpi with TRV were sprayed with 100 μM SA and 100 μM AIP. Each hormone treatment was repeated three times, with the deionized water treatment serving as the control.

### Reverse transcription-quantitative PCR assay

In this assay, total RNA samples were extracted from different parts of petunia plants, and purified to avoid DNA contamination as previously described [46]. Reverse transcription was performed to synthesize cDNA using a HiScript II Q RT SuperMix for qPCR Kit (Novogene Biotech, Jiangsu, China). The synthetic cDNA was used as a template for the amplification with specific primers of target gene fragments (Supplementary Table S1), and petunia *elongation factor 1α* (*PhEF-1α*) was referred to as an internal control to standardize transcript levels. RT-qPCR analysis was performed using the SYBR Premix Ex Taq II (Takara, Otsu, Shiga, Japan). A Roche LightCycler480 instrument (Roche Diagnostic, Basel, Switzerland) was used to run the reactions. This experiment was carried out using three biological replicates. Relative transcript levels were calculated and analyzed following a previously described method [112].

### Virus-induced gene silencing assay

To form the VIGS constructs, a 330-bp fragment of *PhWRKY30* and a 302-bp fragment of *PhPAL1* were amplified using specific primers with *Sac*I and *Xba*I sites, respectively (Supplementary Table S1). The *PhWRKY30* fragment was ligated into the corresponding sites of TRV2-GFP and TRV2-EV, while the *PhPAL1* fragment was only introduced into TRV2-EV vector. The inoculum was prepared, and the plasmids were transformed according to a previous description [113]. *Agrobacterium tumefaciens* strain GV3101 cells transformed with resulting plasmids were cultured in liquid LB medium containing two antibiotics, 50 mg ml^−1^ kanamycin and 50 mg ml^−1^ gentamycin, at 28°C for 2 d. The cultures were collected and suspended in infiltration buffer containing 10 mM MgCl_2_, 10 mM MES salt, and 200 μM acetosyringone, and the concentration was adjusted to a final OD600 = 4.0. The infiltration solution containing TRV1 was mixed with that containing TRV2 constructs in an equal volume. TRV2-GFP and TRV2-EV without any inserts were used as negative controls. The agrobacterial mixture was injected into the petunia leaves using a 3-ml sterile syringe upon removal of needle. This assay was repeated three times, and five plantlets were used for each replicate of infiltration. The fluorescent foci caused by GFP expression were observed via a blue lightemitting diode flashlight with an emission wavelength of 450 nm (3260RB, LUYOR, Shanghai, China). A camera with a bypass filter (LUV-495, LUYOR, Shanghai, China) was used to record the fluorescent signals. The intensity of fluorescent signal was analyzed using ImageJ tool (National Institute of Health, Bethesda, MD, USA).

### Generation of transgenic petunia plants

To obtain transgenic petunia plants with *PhWRKY30* and *PhPAL2b* overexpression, the ORF regions of *PhWRKY30* and *PhPAL2b* were introduced into the pGSA1403 vector between *Xho*I and *Sac*I sites [66, 114]. For the RNAi constructs, a 330-bp fragment of *PhWRKY30* cDNA and a 400-bp fragment of *PhPAL2b* cDNA were amplified using primer pairs harboring *Spe*I-*Asc*I sites and *Bam*HI-*Swa*I sites in sense and antisense orientations, respectively (Supplementary Table S1). The resulting products were introduced into the pGSA1285 vector. The fusion plasmids pGSA1403-*PhWRKY30*, pGSA1403-*PhPAL2b*, pGSA1285-*PhWRKY30*, and pGSA1285-*PhPAL2b* were introduced into *A. tumefacient* strain LBA4404 cells. Young and thick leaves of petunia cultivar ‘Mitchell Diploid’ were cut into 1.0 cm^2^ leaf discs for genetic transformation experiments. The detailed steps of transformation and regeneration have been previously described [45]. The positive transgenic plants of T0 generation were obtained on the medium with addition of 150 mg L^−1^ kanamycin. Self-pollination was performed to propagate transgenic plants until seed harvest of T2 lines. The seeds were then placed on the medium supplemented with the above concentration of kanamycin, and the ones showing 100% survival rates were defined as homozygotes. PCR amplification of the DNA fragment covering CaMV 35S promoter and inserted gene sequence was performed to test the gene introduction into the petunia genome. Transcript abundances of *PhWRKY30* and *PhPAL2b* in WT, RNAi, and OE lines were examined by RT-qPCR.

### Measurement of total chlorophyll content

The content of total chlorophyll in the leaves was measured using a previously described method [115] with minor modifications. In brief, 500 mg of fresh petunia leaves were harvested and cut into thin filaments. Chlorophyll was extracted by immersing the leaf filaments in a 10-ml centrifuge tube filled with dimethyl sulphoxide (DMSO). The tube was incubated with a gentle shaking at 65°C in the dark for 24 h until the samples were completely colorless. After removing leaf filaments, the samples were fixed with DMSO to 10 ml. The absorbance of the DMSO-chlorophyll extraction and blank (pure DMSO) was determined at 645 and 663 nm, respectively. Three biological replicates were used for each measurement, and the mean value was taken for the calculation of total chlorophyll content.

### Measurement of jasmonic acid, SA, and BR contents

The extraction and detection of jasmonic acid (JA), SA, and BR were conducted as previously described [116, 117] with some small changes. Briefly, 500 mg of petunia leaves were ground into powder in liquid nitrogen. The samples were transferred into an extracting solution composed of 80% methanol and 1 mM butylated hydroxytoluene, which were fully mixed using a rotary shaker. After that, the mixed samples were centrifuged at 5000 rpm for 20 min at 4°C. The resulting supernatant was enriched with an Elut C18 extraction column. The samples were then re-solubilized with 300 μl of 0.1% formic acid-methanol solution and further centrifuged at 13 000 rpm for 3 min. The supernatant was extracted for HPLC-ESI-MS/MS analysis. The reference standards for all tested hormones here were obtained from Sigma-Aldrich (St Louis, MO, USA). Each measurement was conducted in triplicate.

### Dual luciferase assay

The constructs were prepared following a previous description [45]. For the effector construct, the ORF region of *PhWRKY30* was ligated into the *Eco*RI-*Kpn*I sites of pGreenII62-SK vector. For the reporter constructs, the promoter sequences of *PhADT1* and *PhNPR1* were introduced into the *Sal*I-*Bam*HI sites of pGreenII0800-LUC vector, whereas the promoter regions of *PhPAL1* and *PhPAL2b* were introduced into the *Sal*I-*Pst*I sites. The empty pGreenII62-SK and pGreenII0800-LUC vectors were referred to as the controls. The resulting plasmids were transformed into *A. tumefacient* strain GV3101 cells. The bacterial fluids transformed with the reporter and effector plasmids were mixed together, and then inoculated into the fully expanded leaves of *N. benthamiana* plants. After 3 days of cultivation, the LUC fluorescence intensity was observed and photographed through the Amersham ImageQuant 800 System (Cytiva, Marlborough, USA) using D-Luciferin (Yeasen, Shanghai, China). The activities of both LUC and REN were analyzed through the Infinite 200 Multifunctional Enzyme Labeler (Tecan, Switzerland) using the Dual-Luciferase Reporter Assay System (Promega, Madison, WI, USA). The activities of protein-DNA interaction are expressed as the ratio of LUC/REN. Three independent experiments were carried out for this analysis.

### Electrophoretic mobility shift assay

The ORF region of *PhWRKY30* was amplified and ligated into the pET-28a vector between *Eco*RI and *Hin*dIII sites. *Escherichia coli* Rosetta (DE3) cells were used for PhWRKY30 protein expression. The expression was initiated through 0.1 mM isopropylthio-β-galactoside and detected using SDS-PAGE electrophoresis. Ultrasonic vibration was used to break 50 ml of bacterial fluids into a transparent and clear solution. It was centrifuged to obtain the supernatant, which was further purified using a ProBond Purification System Kit (Invitrogen, CA, USA). A 36-bp DNA fragment that contains the W-box *cis*-elements (TTGACT and TTGACC) in the promoter of *PhPAL2b* was amplified to synthesize biotin-labeled WT and mutant probes. The WT probe without biotin labeling was used as the competitor of biotin-labeled one. The interaction was carried out at 25°C for 20 min via a LightShift Chemiluminescent EMSA Kit (Thermo Fisher Scientific, MA, USA). The complex separation was conducted via a nondenaturing PAGE method. After transferring to a nylon membrane (0.45 μm), the interaction products were conjugated under UV illumination, and further detected using a Chemiluminescent Nucleic Acid Detection Module Kit (Pierce, Thermo Fisher Scientific, MA, USA). The binding signals were observed and imaged through the Gel Doc XR+ Imaging System (Bio-Rad, Hercules, CA, USA). The protein–DNA binding experiment was repeated three times.

### Measurement of O_2_^−^ and H_2_O_2_ levels

The accumulation of O_2_^−^ and H_2_O_2_ was detected following a previous description [118] with some minor modifications. For O_2_^−^ and H_2_O_2_ measurements, the leaf samples were excised and immersed in 40 mM phosphate buffer (pH = 7.6) containing 0.2 mg ml^−1^ nitroblue tetrazolium and in 60 mM Tris-acetate buffer (pH = 5.0) supplemented with 1.5 mg ml^−1^ 3, 3′-diaminobenzidine under a vacuum, respectively. They were kept at 25°C in the dark for 10 h. The leaves were then destained to remove chlorophyll using 90% ethanol in a water bath at 100°C. The O_2_^−^ content was detected through a superoxide anion assay kit (Solarbio, Beijing, China), while the H_2_O_2_ content was examined through a hydrogen peroxide assay kit (Solarbio, Beijing, China). Three biological replicates were used for the measurement with at least five leaves in each replicate.

## Supplementary Material

Web_Material_uhaf008

## Data Availability

All data supporting the conclusions of this study are present in the paper or Supplementary files.
