# Supplementary material for: PhWRKY30 activates salicylic acid biosynthesis to positively regulate antiviral defense response in petunia
Source: Hortic Res. 2025 Jan 15;12(5):uhaf013. doi: 10.1093/hr/uhaf013 (PMC11966387; doi:10.1093/hr/uhaf013)
Supplement: Web_Material_uhaf008 [file web_material_uhaf008.docx]

**
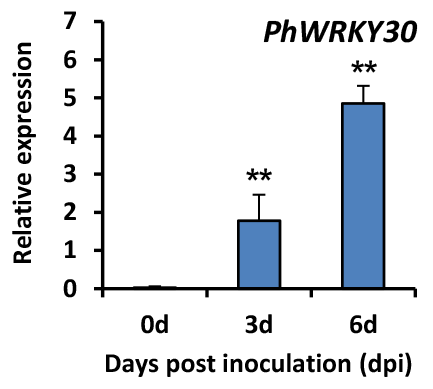
**

**Supplementary Figure S1.** Expression of *PhWRKY30* from Tobacco rattle virus-infected petunia leaf transcriptome data. Transcript levels of *PhWRKY30* in petunia leaves infected with Tobacco rattle virus (TRV, PPK20) at 0, 3, and 6 days post inoculation (dpi) based on the fragments per kilobase of transcript per million mapped reads (FPKM). Error bars represent standard error of the mean from three biological replicates. Asterisks indicate statistical significance as determined by Student’s *t* test (***P* < 0.01).


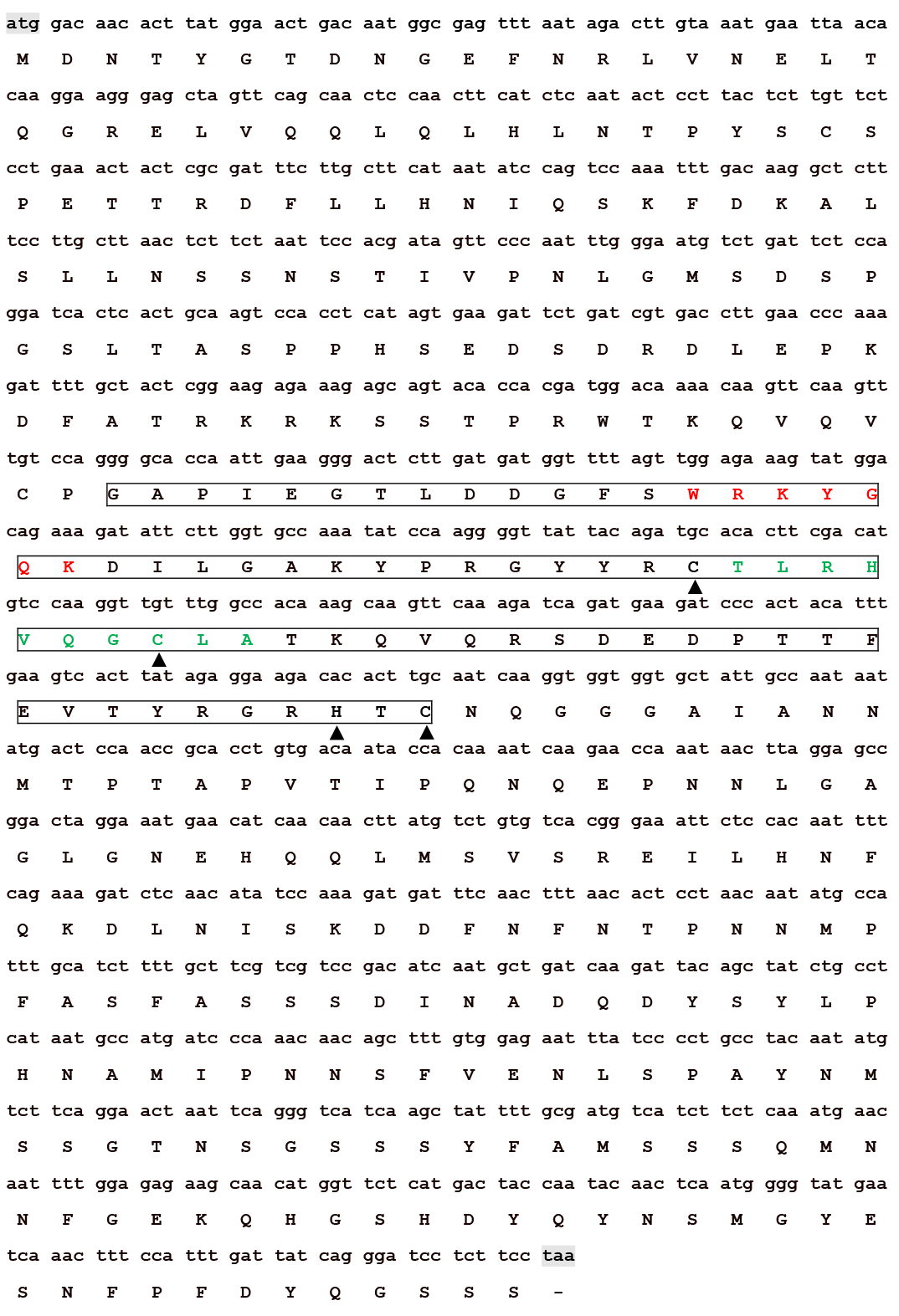


**Supplementary Figure S2.** The nucleotide and deduced amino acid sequences of *PhWRKY30* cDNA. The cDNA sequence contains a 1056-bp coding region encoding a polypeptide of 352 amino acids. The fonts shaded in gray indicate the start and stop codons. The conserved domain of PhWRKY30 is boxed. The WRKY domain and hinge region are highlighted in red and green, respectively. Three cysteines and one histidine within zinc finger region are marked by black solid triangles.

**
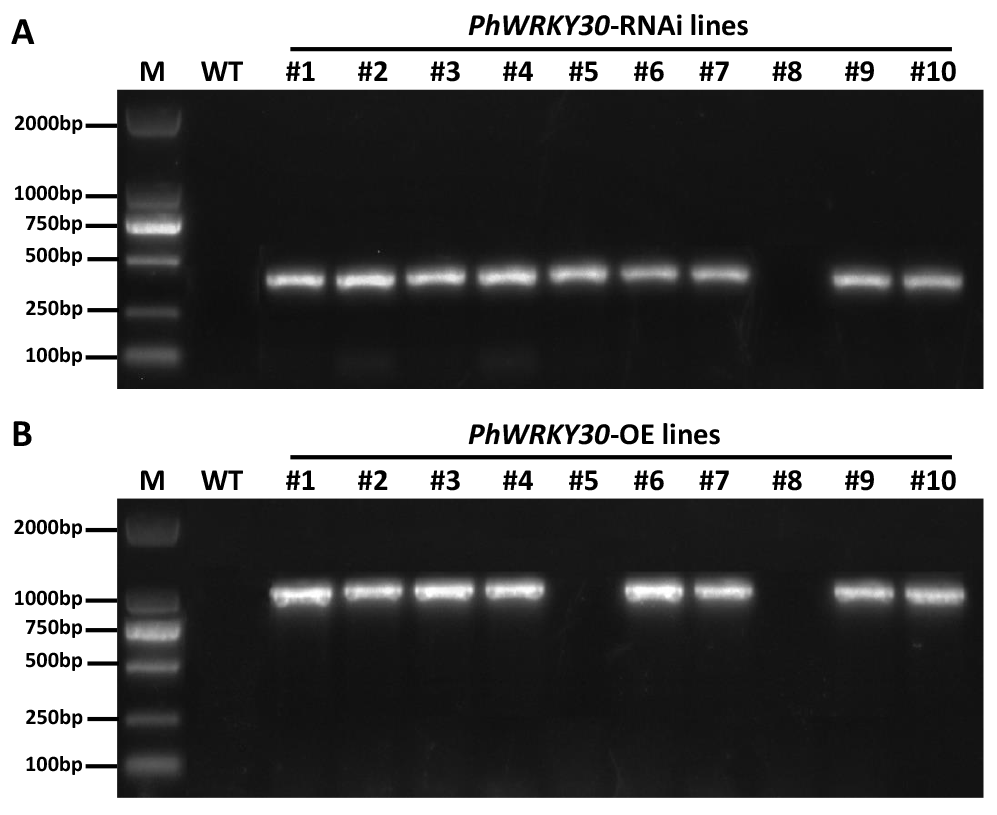
**

**Supplementary Figure S3.** PCR analysis of the transgene insertion into the genome in *PhWRKY30* transgenic petunia plants. Gel bands of the fragments covering CaMV 35S promoter and gene inserts of *PhWRKY30* in the leaves from T0 generation of *PhWRKY30*-RNAi (**A**) and *PhWRKY30*-overexpressing (OE) (**B**) lines. Wild-type (WT) plant was used as the control. Ten candidate lines for RNAi or OE assay were selected for PCR analysis. CaMV 35S promoter is present in the pGSA1285 (RNAi) or pGSA1403 (OE) vector. M, DL2000 DNA marker.


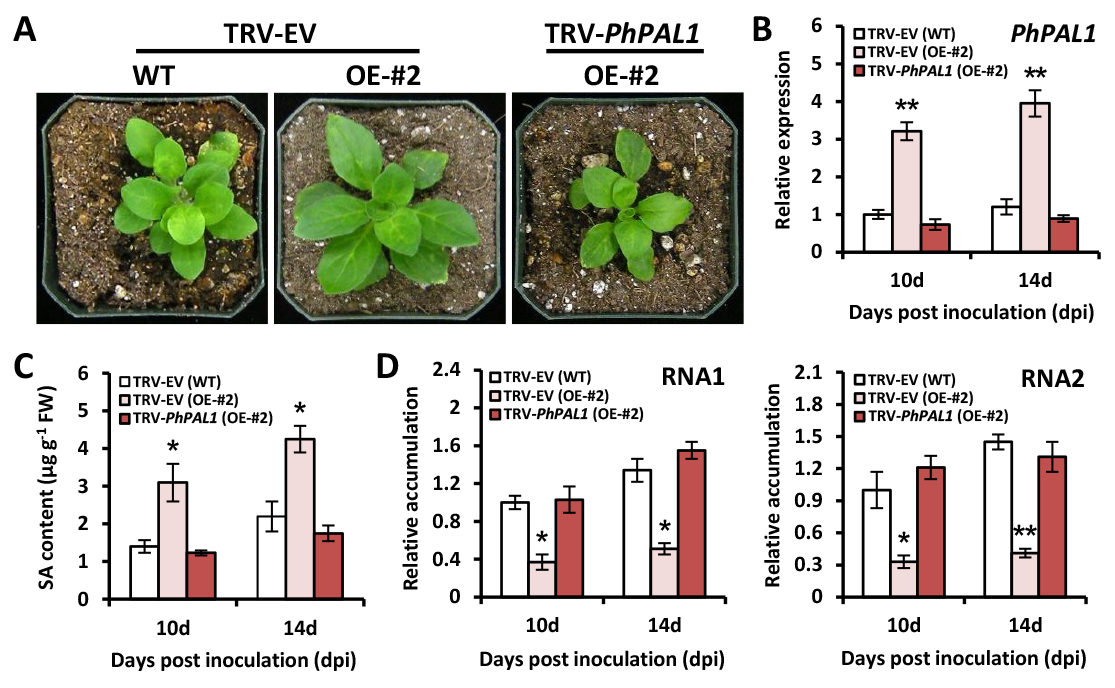


**Supplementary Figure S4.** Silencing of *PhPAL1* inhibits salicylic acid-mediated resistance to Tobacco rattle virus infection in *PhWRKY30*-overexpressing plants. (**A**) Symptoms of wild-type (WT) plants and *PhWRKY30*-overexpressing (OE) line (#2) of petunia cultivar ‘Mitchell Diploid’ inoculated with *Agrobacterium* bearing TRV empty vector (EV) and TRV-*PhPAL1*. The plants at 14 days post inoculation (dpi) were photographed. (**B**) Reverse transcription-quantitative PCR (RT-qPCR) analysis of expression levels of *PhPAL1* in TRV-EV-infected WT and *PhWRKY30*-OE plants and TRV-*PhPAL1*-infected *PhWRKY30*-OE plants at 10 and 14 dpi. (**C**) Content of salicylic acid (SA) and (**D**) relative accumulation levels of TRV RNAs (RNA1 and RNA2) in WT and *PhWRKY30*-OE plants infected with TRV constructs. Expression or accumulation levels were standardized to the reference gene *PhEF1α*. Error bars suggest standard error of the mean from three biological replicates. Statistical significance was determined by Student’s *t* test (**P* < 0.05, ***P* < 0.01) and indicated by asterisks.


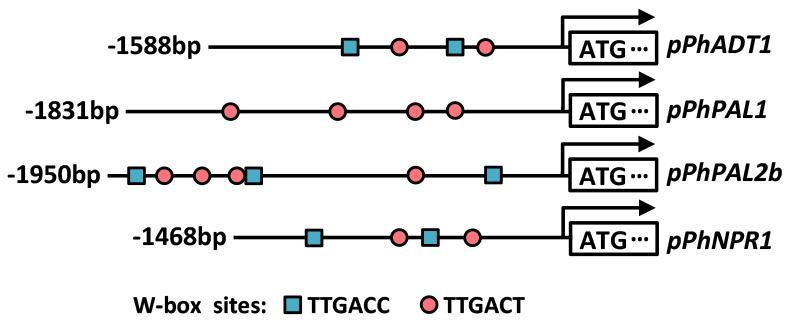


**Supplementary Figure S5.** Graphic representation of W-box motifs in the promoters of *PhADT1*, *PhPAL1*, *PhPAL2b*, and *PhNPR1*. Two W-box *cis*-elements (TTGACC and TTGACT) in various lengths of gene promoters are marked in blue squares and red circles. *pPhADT1*, *pPhPAL1*, *pPhPAL2b*, and *pPhNPR1*, promoters of *PhADT1*, *PhPAL1*, *PhPAL2b*, and *PhNPR1*.


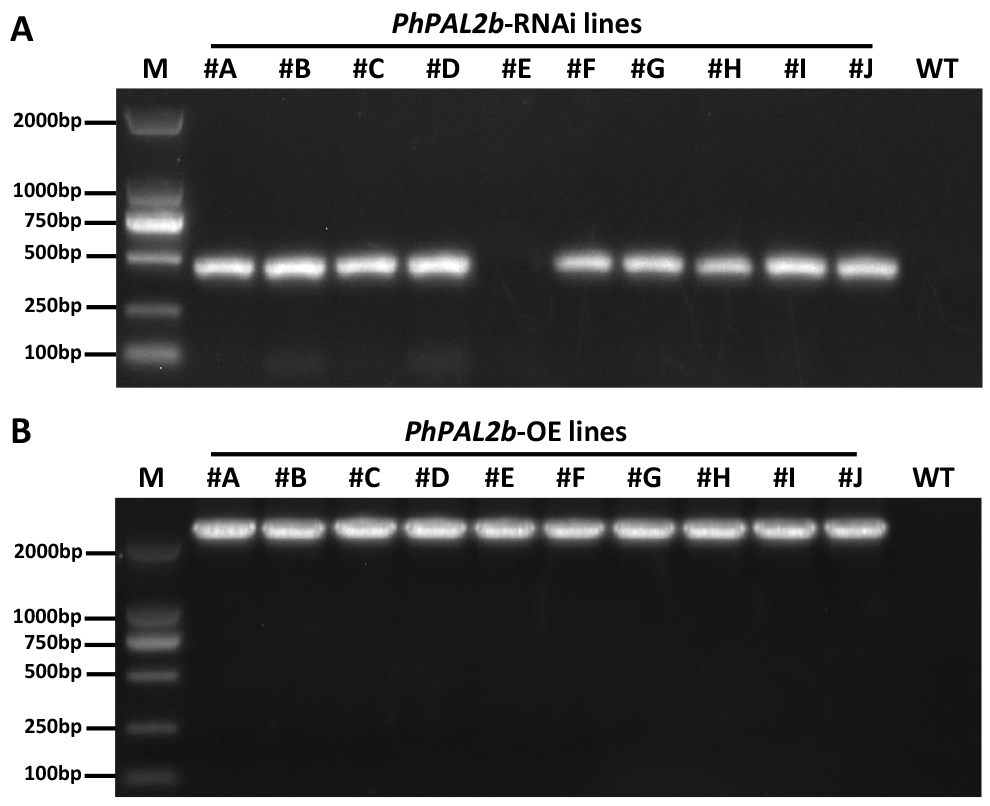


**Supplementary Figure S6.** PCR analysis of the transgene insertion into the genome in *PhPAL2b* transgenic petunia plants. Gel bands of the fragments covering CaMV 35S promoter and gene inserts of *PhPAL2b* in the leaves from T0 generation of *PhPAL2b*-RNAi (**A**) and *PhPAL2b*-overexpressing (OE) (**B**) lines. Wild-type (WT) plant was used as the control. Ten candidate lines for RNAi or OE assay were selected for PCR analysis. CaMV 35S promoter is present in the pGSA1285 (RNAi) or pGSA1403 (OE) vector. M, DL2000 DNA marker.


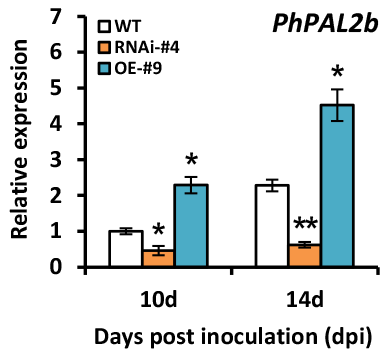


**Supplementary Figure S7.** Expression of *PhPAL2b* in Tobacco rattle virus-infected *PhWRKY30* transgenic petunia plants. Reverse transcription-quantitative PCR (RT-qPCR) analysis of expression levels of *PhPAL2b* in the leaves from wild-type (WT), *PhWRKY30*-RNAi (#4), and *PhWRKY30*-overexpressing (OE) (#9) transgenic lines of petunia cultivar ‘Mitchell Diploid’ inoculated with Tobacco rattle virus (TRV, PPK20). The leaf samples at 10 and 14 days post inoculation (dpi) were harvested for expression analysis. *PhEF1α* was used as the reference gene for expression analysis. Error bars represent standard error of the mean from three biological replicates. Asterisks indicate statistical significance as determined by Student’s *t* test (**P* < 0.05, ***P* < 0.01).


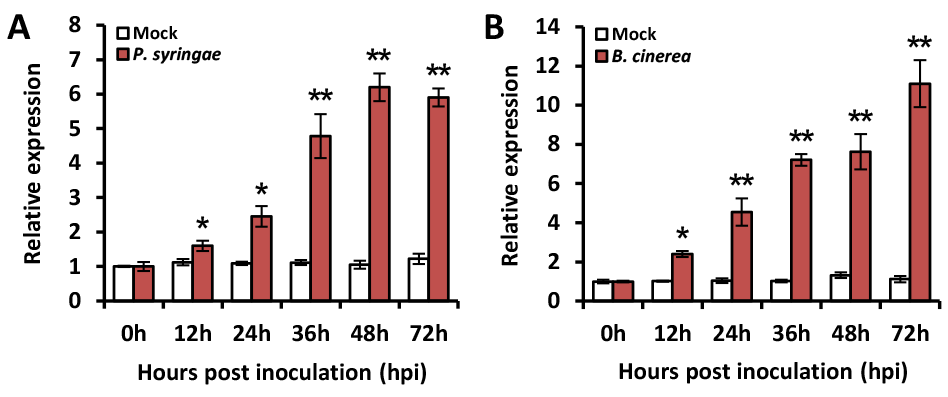


**Supplementary Figure S8.** Expression of *PhWRKY30* in petunia leaves infected with *Pseudomonas* *syringae* and *Botrytis* *cinerea*. Reverse transcription-quantitative PCR (RT-qPCR) analysis of expression levels of *PhWRKY30* in the leaves from petunia cultivar ‘Mitchell Diploid’ plants at different hours post inoculation (hpi) with *P. syringae* pv. *tomato* DC3000 (**A**) and *B. cinerea* Pers.:Fr. (**B**). The *P. syringae* solution containing 10 mM Mg_2_SO_4_ supplemented with 0.025% Silwet77, and the *B. cinerea* conidial suspension containing 10 mM glucose and 6.7 mM K_3_PO_4_ were applied to the leaves of 4-leaf-stage plants. The inoculation with deionized water was used as mock control. A 90% relative humidity was maintained to ensure an effective inoculation. *PhEF1α* was used as an internal control. Error bars represent standard error of the mean from three biological replicates. Asterisks indicate statistical significance as calculated by Student’s *t* test (**P* < 0.05, ***P* < 0.01).


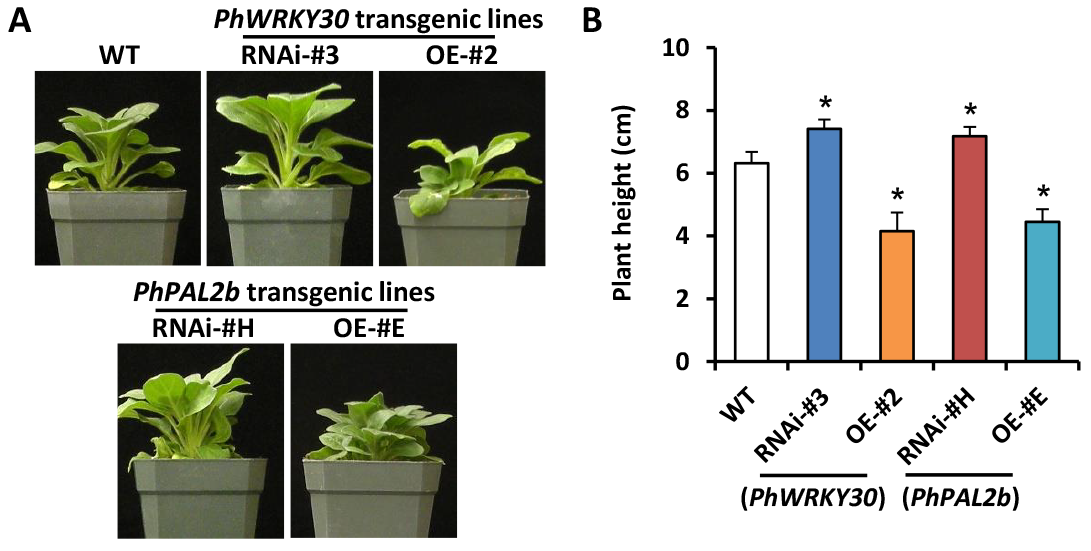


**Supplementary Figure S9.** Effect of RNAi silencing and overexpression of *PhWRKY30* and *PhPAL2b* on petunia plant growth. (**A**) Representative phenotypes of wild-type (WT), *PhWRKY30*-RNAi (#3), *PhWRKY30*-overexpressing (OE) (#2), *PhPAL2b*-RNAi (#H), and *PhPAL2b*-OE (#E) transgenic lines of petunia cultivar ‘Mitchell Diploid’. (**B**) Plant height of WT and transgenic petunia plants with RNAi silencing and overexpression of *PhWRKY30* and *PhPAL2b*. Photographs were taken and plant heights were measured at 3 weeks post germination. Error bars represent standard error of the mean from three biological replicates. Asterisks indicate statistical significance as determined by Student’s *t* test (**P* < 0.05).

**Supplementary Table S1.** Specific primers used for plasmid construct and expression analysis.

| Gene name | Forward primer (5’-3’) | Reverse primer (5’-3’) | Use |
| --- | --- | --- | --- |
| *PhWRKY30* | CATATGATGGACAACACTTATGGAA | GTCGACTTAGGAAGAGGATCCCTGA | Transactivation assay |
| *PhWRKY30* | GAGCTCAGCCGGACTAGGAAATGAACA | TCTAGAGAACCATGTTGCTTCTCTCCA | VIGS assay |
| *PhWRKY30* | ACTAGTGGCGCGCCAGCCGGACTAGGAAATGAACA | GGATCCATTTAAATGAACCATGTTGCTTCTCTCCA | RNAi assay |
| *PhWRKY30* | CTCGAGATGGACAACACTTATGGAA | GAGCTCTTAGGAAGAGGATCCCTGA | Overexpression assay |
| *PhWRKY30* | TTCATTTCATTTGGAGAGGACAC (35S promoter) | GAACCATGTTGCTTCTCTCCA | PCR verification of RNAi insert |
| *PhWRKY30* | TTCATTTCATTTGGAGAGGACAC (35S promoter) | TTAGGAAGAGGATCCCTGA | PCR verification of overexpression insert |
| *PhPAL1* | GAGCTCCTCTTTGGGCTTAATCTCGGC | TCTAGAGTCTGCATCAATGGGTAGTTGC | VIGS assay |
| *PhPAL2b* | ACTAGTGGCGCGCCGGACCCTTTACAAAAGCCTAA | GGATCCATTTAAATGGCAAGAAATTGGAGTTCAGA | RNAi assay |
| *PhPAL2b* | CTCGAGATGGAGTATGCCAATGAAAAC | GAGCTCTTAGCAGAGTGGAAGAGGAGC | Overexpression assay |
| *PhPAL2b* | TTCATTTCATTTGGAGAGGACAC (35S promoter) | GGCAAGAAATTGGAGTTCAGA | PCR verification of RNAi insert |
| *PhPAL2b* | TTCATTTCATTTGGAGAGGACAC (35S promoter) | TTAGCAGAGTGGAAGAGGAGC | PCR verification of overexpression insert |
| *PhWRKY30* | TTCTCCTGAAACTACTCGCGA | TATGAGGTGGACTTGCAGTGA | RT-qPCR assay |
| *GFP* | GAGTACAACTACAACAGCCACAA | CTACTTGTACAGCTCGTCCATG | RT-qPCR assay |
| RNA1 | AATTGTGGATGGTTTGTCTGTG | TCCCAAATTCTCTGTCCTCTTT | RT-qPCR assay |
| RNA2 | GTTACTAGCGGCACTGAATAGA | AGTACTCCCTTGGTTCGTCGTA | RT-qPCR assay |
| *PhCM1* | CACGCAACGGTTGGAAGATAT | GTGAATTCTCTTCGACAGGGC | RT-qPCR assay |
| *PhADT1* | GGCTGCATATTGTGGGAGAAG | GTCCCTTCAGATGCCACAATC | RT-qPCR assay |
| *PhPAL1* | GCCTTGCACTTGTGAATGGTA | CCTTAACATAAGCGCTGCCAT | RT-qPCR assay |
| *PhPAL2a* | AACTCACACATTGCCACAGTC | CCAGTGAGTAATCCGGCAATG | RT-qPCR assay |
| *PhPAL2b* | CCAAAGAAGGTGCAGCTCTAC | ATGCCTGAGTAGCCTTGAAGT | RT-qPCR assay |
| *PhNPR1* | AGGGCATTGGACTCTGATGAT | TGAAGCACCGTGTATCCTCTT | RT-qPCR assay |
| *PhPR1* | TTGACGTTCACAATAAGGCCC | AATAAGGCTTTTCGTCCACCC | RT-qPCR assay |
| *PhWRKY30* | GAATTCATGGACAACACTTATGGAA | GGTACCTTAGGAAGAGGATCCCTGA | Dual luciferase assay |
| *PhADT1* | GTCGACTGTTGGGTTTATTGATTAGGA | GGATCCGGTTCAATGTACCAATACCAA | Dual luciferase assay |
| *PhPAL1* | GTCGACAACGTCGTGTTGAACTTGTCT | CTGCAGTGGATCAAGAAAATGTTTATT | Dual luciferase assay |
| *PhPAL2b* | GTCGACATAGTAATTAAAGAATTTATA | CTGCAGTGCTAGTGCTAAAAGAACAAG | Dual luciferase assay |
| *PhNPR1* | GTCGACTGGAATGTAAGACAAGGGTGG | GGATCCTCGCTTTACATTCGCTTTGTC | Dual luciferase assay |
| *PhWRKY30* | GAATTCATGGACAACACTTATGGAA | AAGCTTGGAAGAGGATCCCTGATAA | EMSA assay |
| *TMV-CP* | TCAGTTCGTGTTCTTGTCATCAG | TCTAGTGTCGAATGCACCTAACA | RT-qPCR assay |
| *PhRbohD* | CGACTTGGATGTACTTGGCTG | GAGACTGCAGCACAATTGACA | RT-qPCR assay |
| *PhRbohF* | CTGGACTCAAGAACTCAAGCG | TCCAATGCCAAGACCAACAAG | RT-qPCR assay |
| *PhSOD* | TCAAGATGGAGATGGCCCTAC | CGACTGTGATGTTGCCAAGAT | RT-qPCR assay |
| *PhAOX* | TGGACTGTTAAGGCTCTTCGT | GCTTTGATCCATCCACCACTC | RT-qPCR assay |
| *PhGPX* | ACAAAGATCAAGGGCTGGAGA | CGATCAACAGCCTTCCCATTC | RT-qPCR assay |
| *PhCAT* | CGTCTTGGGCCAAACTATCTG | AGGAGGAATTGGGTACTGCTC | RT-qPCR assay |
| *PhRDR1* | TCATGATGTCACAATTGAGGAAG | TTCGGAGTGATAGGTTGTCTTGT | RT-qPCR assay |
| *PhRDR2* | CACAAGAATTGGAAATCAAGAGG | TTAACATTCTCCCAACTCTCCAA | RT-qPCR assay |
| *PhDCL1* | AGCTCTATGAGACATGCCAGAAG | CCTTGTTAGCTGCTCTGTTCAAT | RT-qPCR assay |
| *PhDCL2* | GTTATCAGTTGGAAGCATTGGAG | AGTAGCAGCATCCCAATAATCAA | RT-qPCR assay |
| *PhDCL3* | ATGGATGTTGCAAAGAAGAAGAA | TTCTGATCACCATTCCTCAAAGT | RT-qPCR assay |
| *PhDCL4* | CAGCTCTTCTGTTTGGACAACTT | AAGGTTCATGGCATTTCTCTGTA | RT-qPCR assay |
| *PhAGO1* | TCTATCAAAGTATCCGCCCTACA | CTTGAGATGTTAAACCCGAGATG | RT-qPCR assay |
| *PhAGO2* | ACCCAAGTCTGATCTTCGTATCA | TATCACGAGGAATGGATGAGAGT | RT-qPCR assay |
| *PhEF1α* | CCTGGTCAAATTGGAAACGG | CAGATCGCCTGTCAATCTTGG | RT-qPCR assay |
